# Bacterioplankton seasonality in deep high-mountain lakes

**DOI:** 10.3389/fmicb.2022.935378

**Published:** 2022-09-14

**Authors:** Aitziber Zufiaurre, Marisol Felip, Lluís Camarero, Marc Sala-Faig, Jaanis Juhanson, German Bonilla-Rosso, Sara Hallin, Jordi Catalan

**Affiliations:** ^1^CREAF, Cerdanyola del Vallès, Barcelona, Catalonia, Spain; ^2^Área de Biodiversidad, Gestión ambiental de Navarra-Nafarroako Ingurumenkudeaketa (GAN-NIK), Pamplona-Iruñea, Navarra, Spain; ^3^Departament de Biologia Evolutiva, Ecologia i Ciències Ambientals, Universitat de Barcelona, Barcelona, Catalonia, Spain; ^4^Centre d’Estudis Avançats de Blanes (CEAB), CSIC, Blanes, Catalonia, Spain; ^5^Department of Forest Mycology and Plant Pathology, Swedish University of Agricultural Sciences, Uppsala, Sweden; ^6^Department of Fundamental Microbiology, University of Lausanne, Lausanne, Switzerland; ^7^CSIC, Cerdanyola del Vallès, Barcelona, Catalonia, Spain

**Keywords:** microbial ecology, under-ice ecology, bacteria coexistence, core community, Actinobacteria hgcl_clade, Verrucomicrobia, *Flavobacterium*, *Limnohabitans*

## Abstract

Due to global warming, shorter ice cover duration might drastically affect the ecology of lakes currently undergoing seasonal surface freezing. High-mountain lakes show snow-rich ice covers that determine contrasting conditions between ice-off and ice-on periods. We characterized the bacterioplankton seasonality in a deep high-mountain lake ice-covered for half a year. The lake shows a rich core bacterioplankton community consisting of three components: (i) an assemblage stable throughout the year, dominated by Actinobacteria, resistant to all environmental conditions; (ii) an ice-on-resilient assemblage dominating during the ice-covered period, which is more diverse than the other components and includes a high abundance of Verrucomicrobia; the deep hypolimnion constitutes a refuge for many of the typical under-ice taxa, many of which recover quickly during autumn mixing; and (iii) an ice-off-resilient assemblage, which members peak in summer in epilimnetic waters when the rest decline, characterized by a dominance of *Flavobacterium*, and *Limnohabitans*. The rich core community and low random elements compared to other relatively small cold lakes can be attributed to its simple hydrological network in a poorly-vegetated catchment, the long water-residence time (*ca.* 4 years), and the long ice-cover duration; features common to many headwater deep high-mountain lakes.

## Introduction

Declining snow and ice cover duration on lakes is expected with increasing climate warming, which can change ecological dynamics in ways that are hard to predict (Sharma et al., 2019). High-mountain lakes are characterized by great snow accumulation on top of the ice, which drastically reduces light penetration and thereby restricts phototrophic growth to the ice-free period (De Senerpont Domis et al., 2013) and a few weeks at the beginning and end of the ice-covered period (Felip et al., 1999b). Pioneering research in high-mountain lakes suggested differentiated under-ice bacterioplankton communities (Pernthaler et al., 1998). More recent observations confirmed that ice-covered bacterioplankton differs from that of ice-free in terms of composition (Aguilar and Sommaruga, 2020) and metabolic functions, drawing attention to the functional importance of the winter period (Vigneron et al., 2019). Highly active and dynamic under-ice bacterioplankton communities have been described (Denfeld et al., 2018), including heterotrophic (Bižić-Ionescu et al., 2014) and chemoautotrophic microorganisms (Boyd et al., 2011; Achberger et al., 2019). Changes in precipitation and temperature can significantly alter ice-cover dynamics, the bacterioplankton communities, and the whole ecosystem.

Studies of a large number of lakes over hydrological networks have shown that bacterial communities inhabiting lakes are composed of core taxa, whose composition responds to in-lake environmental conditions, and a large fraction of mostly rare taxa, whose presence appears to be locally random and related to the characteristics of the downstream transport in the hydrologic network rather than lake conditions (Niño-García et al., 2016). In an alpine context, bacterial communities in headwater lakes with small, poorly vegetated catchments should show less influence of the hydrologic network and richer core communities. The relevance of lake size in establishing contrasting aquatic communities in high-mountain lakes was shown in a study across European ranges, analyzing the distribution of several relevant groups from protists to insects (Catalan et al., 2009). The lake size effect was not continuous but clearly showed a remarkable ecological threshold at *ca.* 2 ha lake size (or 20-m depth). In shallow lakes, light reaches the bottom throughout the lake; it can be said that the littoral extends to the whole lake, and water residence time is short (<1 year); the water column community can be “washout” during thaw and spring mixing. Most bacterioplankton studies on high-mountain lakes have been carried out in these shallow-type lakes. Less is known about deep lakes with a long water residence time and a profound hypolimnion with physical and chemical characteristics similar to those of the ice-cover period, a “habitat” lacking in shallow lakes. Therefore, we expected deep lakes to show rich bacterioplankton core communities responding to a higher hydrological stability, although in fluctuating environmental conditions throughout the year. Indeed, we hypothesized that the contrasting environmental conditions between the ice-on and ice-off periods should foster overall bacterial diversity by facilitating bacterial coexistence.

The bacterial core community can be seen as a stable non-equilibrium coexistence of many taxa in a fluctuating environment (Chesson, 2000), in which environmental seasonality might play a central role in shaping the community (Mathias and Chesson, 2013; Zufiaurre et al., 2021). According to the theory, the taxa coexistence is based on compensating fitness advantage by niche differentiation. The long-term coexistence of taxa with high differences in maximum growth capacity is only possible if the taxa show highly differentiated niches in an environment of fluctuating conditions. Applying the concept to freezing deep high-mountain lakes, the bacterioplankton core community could be expected to be always integrated by two main types of assemblages. Taxa with high growth capacity should show strong seasonality, simplifying either growing during the ice-covered or ice-free periods, for instance. At the same time, taxa with relatively low growth capacity do not require such strong niche differentiation, which in the temporal domain can be translated into showing no seasonality or, in other words, being resistant to all environmental fluctuations.

Based on this hypothesis, we performed a detailed water column profile 1-year survey of the bacterioplankton community of a deep high-mountain lake (ice-covered for about 6 months per year). We used 16S RNA gene sequencing to characterize the bacterial communities and evaluated their spatial (i.e., depth) and seasonal fluctuations. To discern between taxa showing seasonality and taxa with non-significant fluctuations across the year, we applied a procedure in which the partition of the whole set of samples in classes depicting the seasonality emerges from the patterns of change in the bacterial community (Catalan et al., 2009), that is, “seasons” are no imposed by aprioristic environmental considerations.

## Materials and methods

### Site and sampling

**Figure 1:**
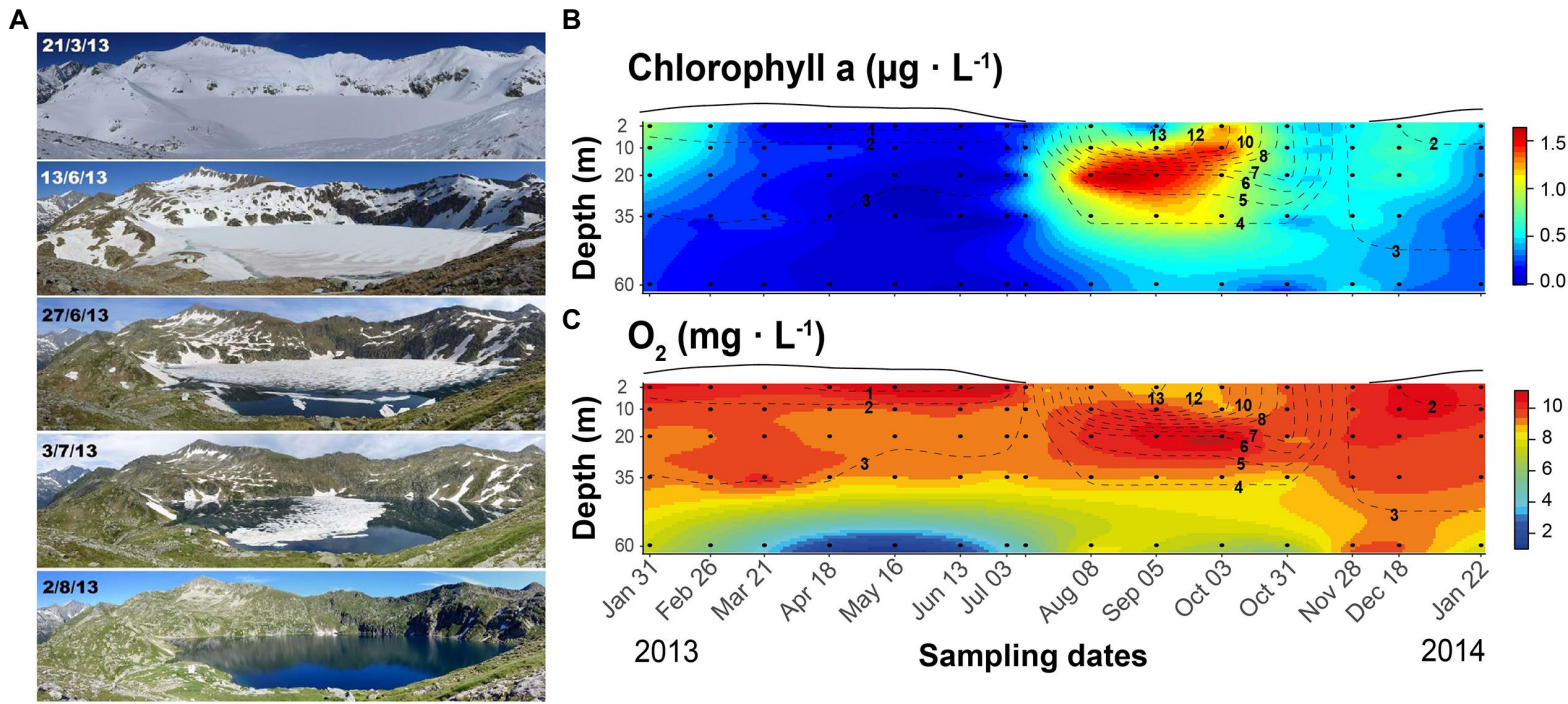
Seasonal changes in Lake Redon, Pyrenees. **(A)** Illustration of the snow and ice cover melting period. Photographs: Marc Sala-Faig. **(B)** Seasonal and depth variation of chlorophyll-a and **(C)** oxygen. Black circles indicate sampling points and dashed lines the isotherms (°C). The line above each graph indicates the snow and ice-cover thickness in arbitrary units.

The study was conducted in lake Redon, an ultraoligotrophic high-mountain lake located at 2232 m a.s.l. in the Pyrenees (42^o^ 38′ 33″ N, 0^o^ 36′ 13″ E), with an area of 0.24 km^2^, maximum and mean depth of 73 and 32 m, respectively, and water residence time of about 4 years. The lake is covered by ice and snow for 6–7 months (Figure 1) and stratifies during the ice-off period. Phytoplankton primary production usually peaks during spring and autumn mixing periods, and a deep chlorophyll maximum develops during summer stratification when the photic zone extends beyond the seasonal thermocline (Felip and Catalan, 2000).

Sampling was performed every 4 weeks, from January 2013 to January 2014. Vertical temperature profiles were performed at the deepest part of the lake at 6-cm intervals using a 6,920 V2 probe (YSI). During the ice-free season, water transparency was estimated with a Secchi disk (z_sd_) and relative surface irradiance (Iz) at depth (z) by assuming an extinction coefficient k = 1.7/z_sd_ (French et al., 1982). Water samples (7 l) were obtained at 2, 10, 20, 35, and 60-m depths. A subsample for oxygen determination with the Winkler method was carefully filled and fixed, minimizing air contact. Then, other subsamples were filtered through a 250-μm pore mesh to remove large zooplankton. For chemical analyses, water was filtered through a precombusted (5 h, 450°C) glass fiber filter (GF/F, Whatman), and the filter was used for particulate matter analyses. For chlorophyll-a (Chla) analysis, 0.5 l was similarly filtered, and the filter was wrapped in aluminum foil and frozen immediately to prevent degradation. Finally, a subsample for bacterial community analysis (400 ml) was filtered through a 0.2 μm pore size polycarbonate filter (47 mm Ø) until saturation and preserved with lysis buffer (40 mM EDTA, 50 mM Tris, pH 8.3, and 0.75 M sucrose) at −80°C until DNA extraction.

### Chemical analyses

Water samples were transferred to the laboratory in bottles without air space to avoid gas exchange that could modify the *in situ* pH, measured with a fast response, low ionic strength electrode (Crison-Hach 5,224). Total dissolved phosphorus (TDP), soluble reactive phosphorus (SRP), ammonium (NH_4_^+^), nitrite (NO_2_^−^), and dissolved reactive silica (DRSi) were determined by colorimetry using a segmented flow autoanalyzer (AA3HR, Seal/Bran+Luebbe, Rijen, The Netherlands). For TDP and SRP, the method (Bran+Luebbe G-175-96) was based on Murphy and Riley (1962). For TDP, filtered samples were previously digested by the acid persulfate oxidation method (Grasshoff et al., 1983). NH_4_^+^ was determined by the blue indophenol method (B+L G-171-96) and NO_2_^−^ by the Griess reaction (B+L G-173-96). DRSi was determined by molybdic-silicate reduction to heteropoly blue (B+L G-171-96). Nitrate (NO_3_^−^) and sulfate (SO_4_^2−^) were measured by capillary electrophoresis (Quanta 4,000, Waters). Dissolved inorganic nitrogen (DIN) was calculated as the sum of NO_3_^−^, NO_2_^−^, and NH_4_^+^. Total nitrogen (TN) was determined as nitrate after sample autoclave oxidation adding an alkaline sodium persulfate solution containing boric acid and sodium hydroxide (Grasshoff et al., 1983). Dissolved organic nitrogen (DON) and dissolved organic phosphorus (DOP) were estimated by the difference between the measured total and the inorganic forms. Oxygen was determined by Winkler titration (Grasshoff et al., 1983). Dissolved organic carbon (DOC) was estimated by catalytic combustion and infrared spectrometric detection (TOC-5000 Shimadzu, Tokyo, Japan). CO_2_ was determined from total dissolved inorganic carbon by infrared spectrometry and alkalinity titration. Particulate carbon and nitrogen (PC and PN) were determined using an elemental analyzer (Thermo EA 1108 CHNS-O, Carlo Erba) and particulate phosphorus (PP) by acid persulfate wet oxidation followed by SRP analysis. Chla was extracted in 90% acetone with an ultrasonic homogenizer (Sonopuls GM70, Delft, The Netherlands; 50 W, 2 min); extracts were centrifuged (4 min at 3,000 rpm, 4°C) and filtered through a Whatman Anodisc 25 (0.1 μm), and quantified by spectrophotometry (Jeffrey and Humphrey, 1975).

### DNA extraction and 16S rRNA gene quantification

DNA was extracted using the phenol/chloroform method (Rusch et al., 2007) without the final CTAB step and quantified using a Qubit fluorimeter (Thermo Fisher Scientific Inc.). Quantitative real-time PCR (qPCR) was used to quantify the bacterial 16S rRNA gene using the primers 341F and 534R (López-Gutiérrez et al., 2004; Supplementary Table S1). Two 15 μl duplicate reactions were conducted on different runs using a Bio-Rad CFX Connect Real-Time System (Bio-Rad Laboratories, CA, United States), and the average gene copy number per sample was calculated. Each reaction contained 3 ng of DNA, 1 mg of BSA (New England Biolabs, MA, United States), 1× Bio-RadiQ™ SYBR^®^ Green Supermix (Bio-Rad Laboratories), and the primers (0.5 μmol L^−1^). Standard curves within the range were obtained by serial dilutions of linearized plasmids containing a cloned fragment of the 16S rRNA gene.

### 16S rRNA gene sequencing and bioinformatic analysis

The diversity, structure, and composition of the bacterial communities were determined by sequencing the V3–V4 hypervariable region of the 16S rRNA gene. Amplicon libraries for each sample were generated using a two-step Polymerase Chain Reaction (PCR) protocol (Berry et al., 2011). The first step was carried out in duplicate per sample and consisted of a 15 μl reaction blending 10 ng of the sample with 2X Phusion PCR Master Mix, 0.25 μM of each 16S rRNA universal primers Pro805R and Pro341F (Takahashi et al., 2014) and 0.5 μg μL^−1^ BSA. Thermal cycling consisted of 25 cycles using the Applied Biosystems 2720 thermal cycler (Supplementary Table S1). Replicate products were pooled and purified using the Agencourt AMPure kit. In the second step, 3 μl of the purified product were mixed with 0.2 μmol L^−1^ of each Nextera adapter sequence and similar reagent concentrations as in the first step. This step was also performed in duplicate reactions (30 μl) for each sample. The duplicate products were pooled and purified following the Agencourt AMPure XP method, quantified using a Qubit fluorimeter (Thermo Fisher Scientific Inc.), and equimolarly pooled into the library pool. The product was inspected on 1.0% SB gel before Illumina Miseq (2 × ≥250 bp) sequencing at Microsynth (Switzerland).

Reads were trimmed, and quality-checked with FastQC^1^ and FastX Toolkit,^2^ and pair-end reads were merged using PEAR (Zhang et al., 2014). Reads were dereplicated and clustered at 97% identity with VSEARCH (Rognes et al., 2016). Chimeras were identified sequentially using a *de novo* approach and the ‘gold’ reference database implemented in VSEARCH. For the OTU table construction, clusters with less than five reads were removed, and relative abundances were calculated by mapping back the quality-filtered reads at 97% identity. The OTU table comprised 749 OTUs that recruited 3,956,971 quality-filtered reads. Representative sequences from each OTU were classified using the SILVA (v.138) database (Quast et al., 2013) with qiime2-2021.2 (Bolyen et al., 2019). OTUs classified as chloroplast or mitochondria were removed. A higher proportion of chloroplasts did not coincide with 16S rRNA gene abundance peaks. Rarefying to 28,098 sequences per sample was performed with the *phyloseq* R package (McMurdie and Holmes, 2013), resulting in a final table of 651 OTUs. The sequences are available in the NCBI GenBank MZ245731-MZ246381.

### Data analyses

A general appraisal of the bacterial communities variation was performed by a Principal Components Analysis (PCA) using the Hellinger distance (Legendre and Gallagher, 2001), which revealed the seasonal sample sequence and depth differences. However, the core of our data analyses was the definition of a seasonal partition and selection of indicator taxa. According to the coexistence theory (Li and Chesson, 2016), we expected that the community variation would include assemblages showing seasonal differences in their populations, responding to changing resources and conditions, and assemblages without such marked seasonality, which could be fitted to deal with the fluctuating conditions across seasons and space. To differentiate between these two types of taxa, we performed an optimal partition in clusters of the entire sample set that maximized the sum of an indicator of seasonality for each taxon (Catalan et al., 2009). Specifically, we used k-means and the Hellinger distance for clustering the samples and IndVal as taxon indicator (Dufrene and Legendre, 1997). IndVal, the indicator value of a taxon for a given partition cluster, has two components, specificity and fidelity. Specificity is the tendency of a taxon to be found in a single cluster of the classification. Fidelity is the tendency of the taxon to be present in many of the samples belonging to that cluster. We applied a random reallocation procedure of the taxon abundance among samples to test the IndVal significance (*p* < 0.05) using the *indicspecies* R package (De Caceres and Legendre, 2009). To obtain the optimal partition with more taxa significantly indicative of seasonality, we performed successive *k*-means classification of an increasing number of clusters, starting in *k* = 2. In each step, we evaluated the sum of the significant OTU’s IndVal values, and eventually, the *k* partition with the highest cumulative IndVal was selected as representing the optimal seasonal pattern of the bacterial community. PCA and *k*-means were performed using *vegan* R package (Dixon, 2003). According to this procedure, significant indicator OTUs should show fluctuating populations, with higher population densities under conditions of the seasonal cluster for which they were significant. Conversely, OTUs not significant for any cluster would have either stable populations throughout depth and seasons or show occasional random occurrences; not being part of the core community in this latter case. To limit for spurious effects of multiple tests, we only checked the significance of the highest IndVal value of a taxon in a specific *k*-means classification as recommended by Dufrene and Legendre (1997), and applied the Benjamini and Hochberg's (1995) method to maintain a maximum overall 5% false discovery rate.

Diversity within each cluster was assessed using the exponential of Shannon entropy (Jost, 2006), and significant differences between clusters were tested by ANOVA and *post hoc* pairwise comparisons with the HSD Tukey test (Chambers et al., 2017). Linear discriminant analysis (LDA) was performed to relate the OTU assemblage clusters to environmental conditions (Tharwat et al., 2017). In this analysis, all the environmental variables, except pH and ratios, were log-transformed to reduce data skewness, and the *lda* function from the *flipMultivariates* R package was used. No selection of the environmental variables was performed, which can produce some overfitting, because the aim was not to explain clusters by environmental conditions but to characterize the multi-variate environmental conditions in which the clusters occur. Plotting was performed with *ggplot2*, *raster*, *fields*, and *rworldmap* R packages. All the analyses were performed with the R software version 3.4.3 (R Development Core Team, 2020).

## Results

### Lake seasonality

The lake mixing regime was typically dimictic. The ice-covered period extended until early July, with a rapid shift of the ice-cover conditions during late spring and early summer (Figure 1A). After a short period of complete mixing of the water column, the stratification lasted from August to October. Epilimnetic temperature was always <14°C (Figures 1B,C), and the metalimnetic gradient (11–6°C) was located between 10 and 20-m depths. The whole water column mixed again in November, after a progressive thermocline deepening during September and October. The lake surface froze at the end of November, and the inverted weak stratification started, 0–3.8°C in the 60-m water column.

The Chla and O_2_ seasonal fluctuations summarize the lake metabolic changes as indicators of phytoplankton growth and the photosynthesis/respiration balance, respectively. The highest Chla levels occurred between 20 and 35-m depth in August and September, always being <2 μg L^−1^ (Figure 1B). Chla spread all over the mixing layer when the thermocline was deepening, and there was a slight increase at the beginning of the ice-covered period. As soon as heavy snowfall began, Chla declined progressively throughout the under-ice period and remained low (<0.03 μg L^−1^) from March to June. The water column layers above 35-m depth were well-oxygenated throughout the year (>8 mg O_2_ L^−1^). At 60-m depth, the O_2_ concentration was slightly lower than in the upper layers during summer, autumn overturn, and early ice-covered stratification, but much lower during mid and late winter stratification, reaching hypoxia (<2 mg O_2_ L^−1^) from April to June.

The chemical features were relatively stable in most of the water volume, as indicated by the similarity between the sample mean and median values (Supplementary Table S2). The stronger fluctuations were related to the seasonal extremes indicated by Chla and O_2_ fluctuations. Regarding nutrients, phosphorus availability was low compared to nitrogen (Supplementary Table S2), with average DIN levels (~10 μmol L^−1^) 500-fold higher than TDP (~0.02 μmol L^−1^). DOC levels were < 0.5 mg L^−1^. The particulate matter stoichiometry reflected the low P availability compared to carbon and nitrogen, particularly in the summer epilimnion. Despite the low water acid-neutralizing capacity, pH was usually stable and circumneutral (~6.5); pH < 6 only occurred in deep layers at the end of the ice-covered period, and pH > 7 in the Chla maximum during the ice-free period. The deviations were, respectively, related to CO_2_ accumulation and depletion. Sulfate and dissolved silica were always high compared to other nutrients.

### Bacterioplankton abundance and richness

**Figure 2:**
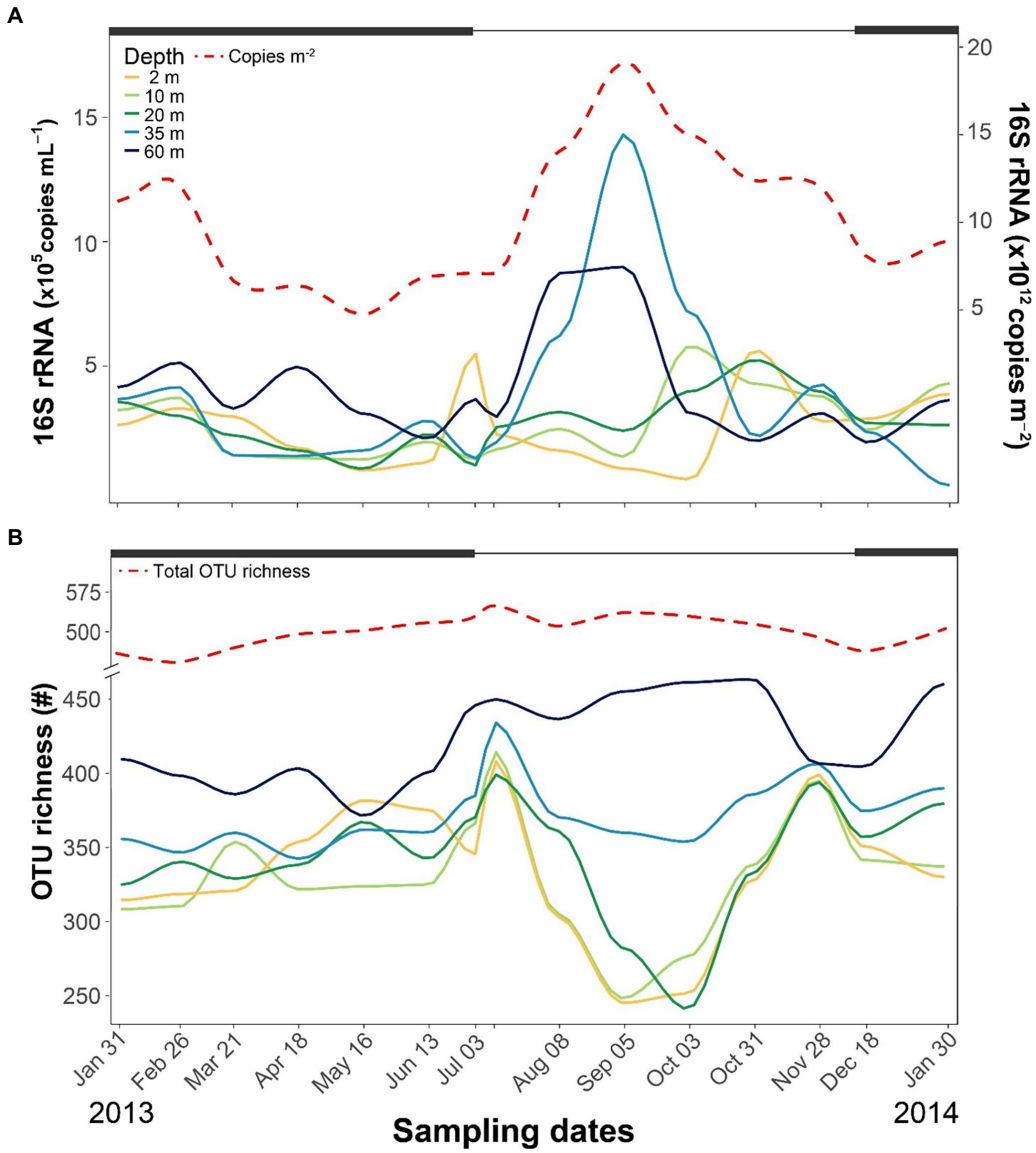
Seasonal fluctuations of bacterioplankton number of 16S rRNA gene copies **(A)** and OTU richness **(B)** at different depths. Thick black lines at the top of each graph indicate periods of ice cover.

Across the year, 16S rRNA genes ranged between 10^5^ and 10^6^ copies mL^−1^, with the highest levels detected in the hypolimnetic layers during summer (Figure 2A). From the surface to 20-m depth, copies of 16S rRNA genes were similar throughout the year (2.6 × 10^5^ ± 1.3 × 10^5^ copies mL^−1^). By contrast, the number peaked at 35-m depth in September and was also higher than average at 60-m depth during summer. The water column-integrated 16S rRNA gene copies peaked in September (~2 × 10^13^ copies m^−2^) during the highest Chla levels.

The identified 651 OTUs comprised 40 classes (Supplementary Table S3) and 147 families (Supplementary Table S4). The dominant classes were Gammaproteobacteria (161 OTUS), Bacteroidia (121), Alphaproteobacteria (109), Actinobacteria (56), Verrucomicrobiae (39), and Acidimicrobiia (20). The OTU richness and seasonal patterns from the surface to 20-m depth were similar, with lower richness during summer stratification and higher during the two mixing periods (Figure 2B). At 35-m depth, richness was similar to the upper layers but without a decline during summer stratification. The highest richness was observed at 60-m depth, particularly during the ice-free period. The OTU richness homogenized across depths during both mixing periods, and the total OTU richness across the entire water column displayed minimal fluctuations over the year.

### Bacterioplankton seasonality

**Figure 3:**
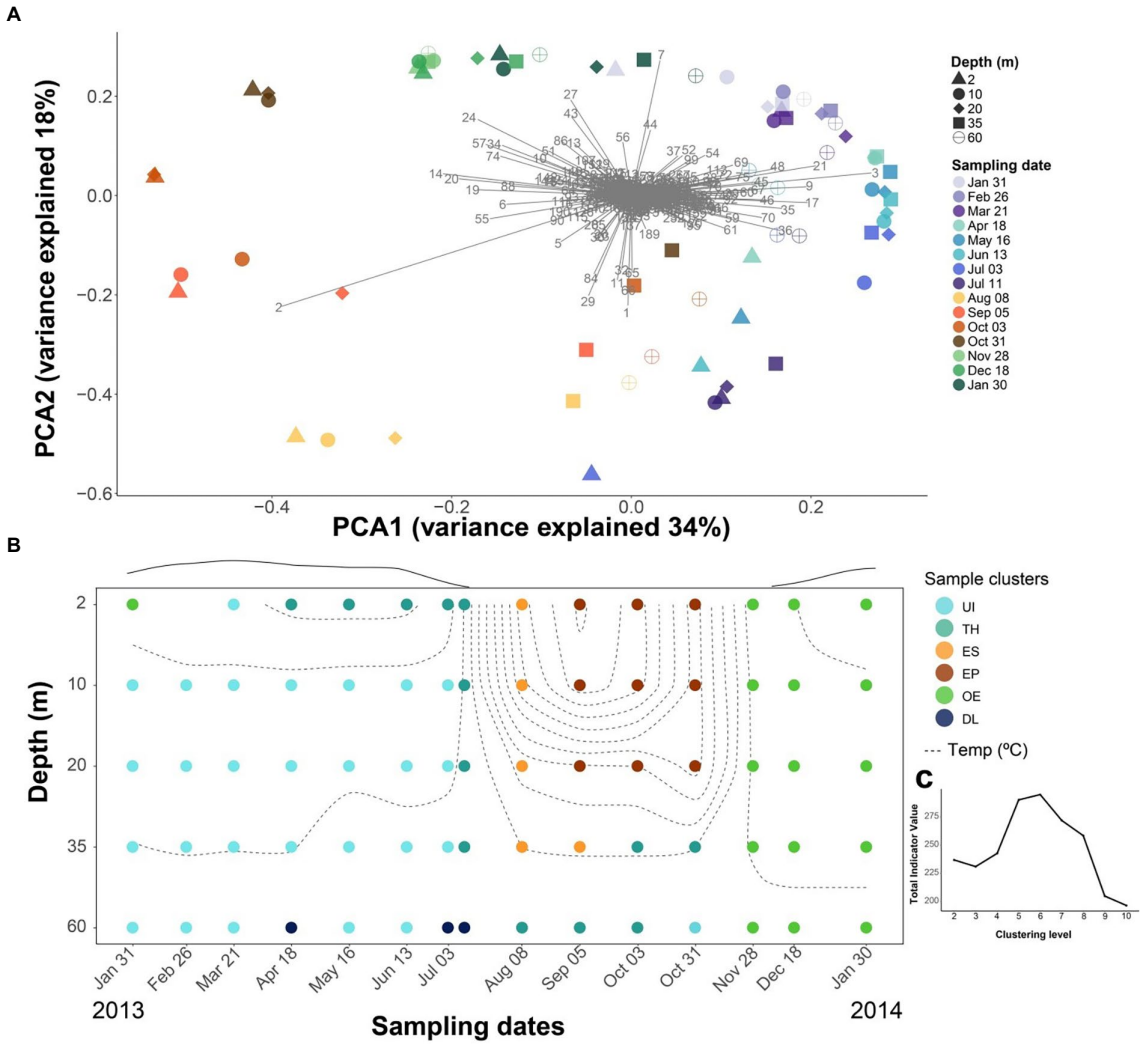
Seasonal difference in bacterioplankton community composition. **(A)** PCA analyses using Hellinger distance of the bacterioplankton OTUs. Numbers refer to OTUs listed in Supplementary Table S4. There is a seasonal trajectory for the upper layers (2, 10, 20-m depth), whereas variation is lower in the deeper layers (35, 60-m depth). **(B)** Temporal depth distribution of the bacterioplankton groups was obtained using k-means clustering with Hellinger distance. Dashed lines denote isotherms. Circles indicate sampling points, and the color corresponds to each of the six clusters: UI (under-ice), TH (thaw and hypolimnion), ES (early stratification), EP (epilimnion), OE (overturn and early under-ice), and DL (deep layers). The line above the graph indicates the snow and ice-cover thickness in arbitrary units. **(C)** The best number of k-means groups was assessed by maximizing the total indicative value (IndVal) of the significant OTUs at each partition.

The community composition showed seasonal and depth patterns, although dissimilarities were more pronounced over time than between depths. The ordination indicated a continuous seasonal shift of the community composition rather than abrupt changes (Figure 3A), with the first axis mainly differentiating the upper layers (2–20-m depth) during the ice-free period from the rest, and the second axis, the differences between spring and autumn overturn samples. There were prominent OTUs associated with summer stratification (OTU-2, *Flavobacterium*), autumn overturn (OTU-24, -27 and -7; Verrucomicrobiae), winter period (OTU-21, Acidobacteria; -3 and -9, CL500-29-marine group; -17, Gemmataceae; -36, Gallionellaceae), and thaw and deep summer layers (OTU-1, *Polaromonas*; OTU-29, *Pedobacter*).

The clustering procedure resulted in an optimal partition of six seasonal clusters (Figures 3B,C), which were named according to their seasonal features as “Under Ice” (UI, 28 samples), “Thaw and hypolimnion” (TH, 13), “Early stratification” (ES, 5), “Epilimnion” (EP, 9), “Overturn and early under ice” (OE, 16), and “Deep layers” (DL, 3). There were 410 OTUs significantly related to one of the clusters (i.e., indicators) and represented, on average, ~80% of the bacterial abundance in the samples. The remaining 241 OTUs did not show fluctuations in abundances through time or space that could be distinguished from random oscillations.

### Environmental conditions of community shifts

**Figure 4:**
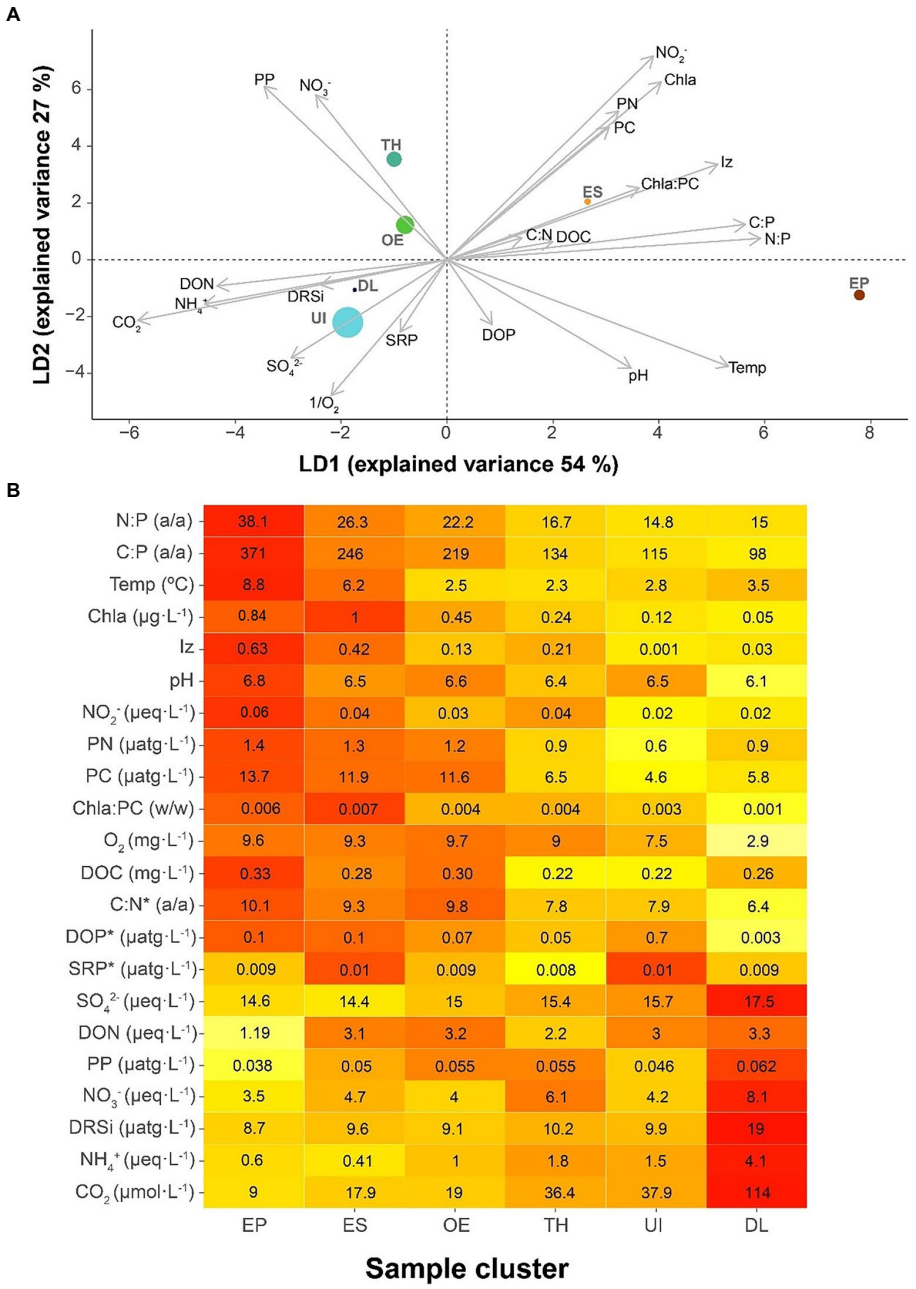
Characteristics of the bacterial seasonal clusters’ environment. **(A)** Bacterioplankton clusters in a discriminant environmental space. Clusters: UI (under-ice), TH (thaw and hypolimnion), ES (early stratification), EP (epilimnion), OE (overturn and early under-ice), and DL (deep layer). The symbol size of each cluster is proportional to the number of samples included. **(B)** Heat map indicating the average environmental conditions for each bacterioplankton seasonal cluster. The average cluster values clusters indicated within the boxes, and colors indicate the relative rank among clusters, from the highest (red) to the lowest (pale yellow). The variables are sorted according to their loading in the first axis of the discriminant analysis (A), with the lowest *p*-values at both extremes and the less relevant variables in the middle. The asterisk (*) indicates no significance in the discriminant analysis.

The environmental conditions discriminating between seasonal bacterioplankton clusters were primarily related to those in the upper layers during summer (Figure 4A), which differentiated EP and ES clusters from the rest. The most conspicuous features of these clusters’ conditions were P-impoverished seston, high irradiance and temperature, and high Chla compared to PC. The second discriminant axis was related to higher nitrate levels, nitrite and particulate organic matter, and low dissolved phosphorus. This situation was characteristic of transition clusters (i.e., TH, ES, and OE).

EP and DL occupied two environmental extremes: EP in conditions of N:P, C:P, temperature, and Chla high values and DL of CO_2_, NH_4_^+^, and DRSi (Figure 4B). These extremes indicate biogeochemical conditions of P-limited primary production and deep-water heterotrophic recycling, respectively. ES, OE, TH, and UI conditions progressively ranged between the extremes.

### Diversity in the seasonal clusters

**Figure 5:**
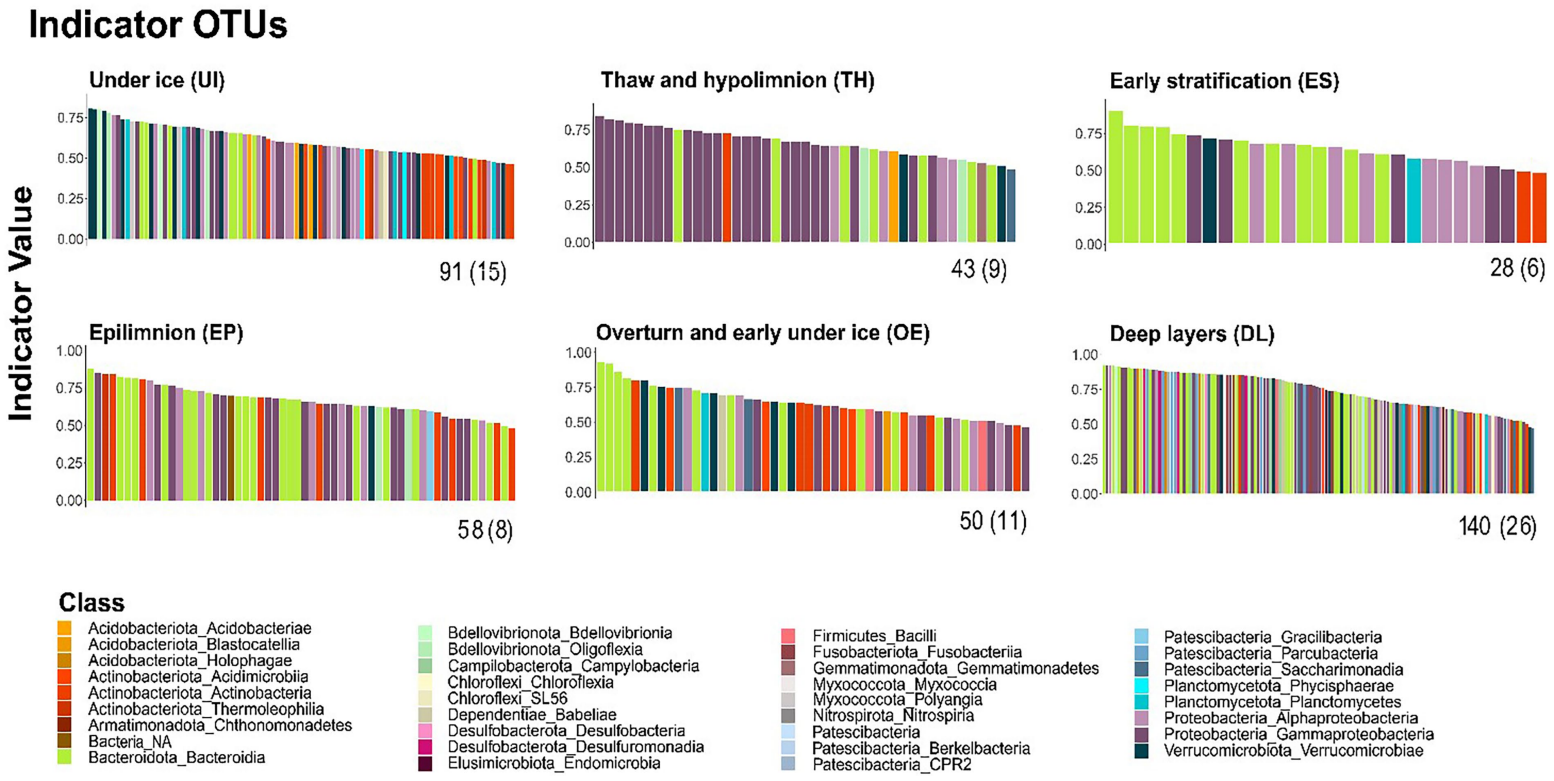
Indicator taxa of seasonal clusters. OTUs are ranked according to their significant indicator values. Colors indicate the taxonomic class. The number of indicator OTUs and classes (parenthesis) are indicated below the *x*-axis.

High-rank taxonomic diversity in each seasonal cluster was elevated, as indicated by the number of taxonomic classes the respective indicator OTUs encompassed (Figure 5; Supplementary Table S3). There was no dominant indicator class except Gammaproteobacteria in TH and Bacteroidia in ES (Figure 5). DL was the most idiosyncratic cluster, comprising 26 taxonomic classes despite the small number of samples. Several classes were exclusively found in this cluster, e.g., Holophagae, Chthonomonadetes, Campylobacteria, Chloroflexia, Desulfobacteria, Desulfuromonadia, Endomicrobia, Fusobacteriia, Myxococcia, Nitrospiria, Berkelbacteria, CPR2, and Parcubacteria. The number of OTUs with a significant indicator value ranged from 28 in the ES cluster to 140 in DL.

The seasonal clusters showed significant differences in diversity (*p* < 0.001). Three groups were apparent (Supplementary Figure S1). The epilimnetic clusters (ES and EP) showed lower diversity per sample, with median exponential Shannon entropy (D) below 20. OE and DL samples showed intermediate diversity within a narrow range around D ~ 30. The highest values were found in UI (D ~ 40) and TH clusters (D ~ 36). This high diversity in TH contrasted with the low variation in high-rank taxonomic levels (Figure 5). These diversity values concerned indicator taxa. Background OTUs always showed lower diversity, with an average D ~ 15.

During the ice-covered period (UI), the most relevant families were Acidimicrobiia Ilumatobacteraceae, Actinobacteria Sporichthyaceae, Verrucomicrobiae (Pedosphaeraceae, and Chthoniobacteraceae), Gammaproteobacteria Comamonadaceae, and Bacteroidia Chitinophagaceae (Supplementary Figure S2A). Bacteroidia, Flavobacteriaceae, and most of the Gammaproteobacteria families (Alcaligenaceae, Comamonadaceae, Methylophilaceae, and Oxalobacteraceae) increased during thawing (TH cluster) following the seasonal shifts, whereas Verrucomicrobiae declined. The bacterial assemblages strictly related to the summer stratification period (ES and EP) showed the lowest number of indicator families (Supplementary Figure S2A) and Bacteroidia Flavobacteriaceae, Gammaproteobacteria Comamonadaceae, and Actinobacteria Sporichthyaceae became prominent. Following the summer stratification, the bacterioplankton community started to recover the previous season’s under-ice composition (Supplementary Figure S2A). A gradual decrease of the most abundant families in ES and EP samples, and recovery of the UI indicator families, characterized the overturn (OE) samples. Verrucomicrobiota was the most favored phylum; the prominent families started to increase at the end of summer, reaching their highest relative abundances in the OE samples. DL cluster showed an increased number of families as expected from the high richness in classes, and the abundance of Gammaproteobacteria was noteworthy, especially Methylomonadaceae.

### Bacterial assemblage not displaying seasonality

**Figure 6:**
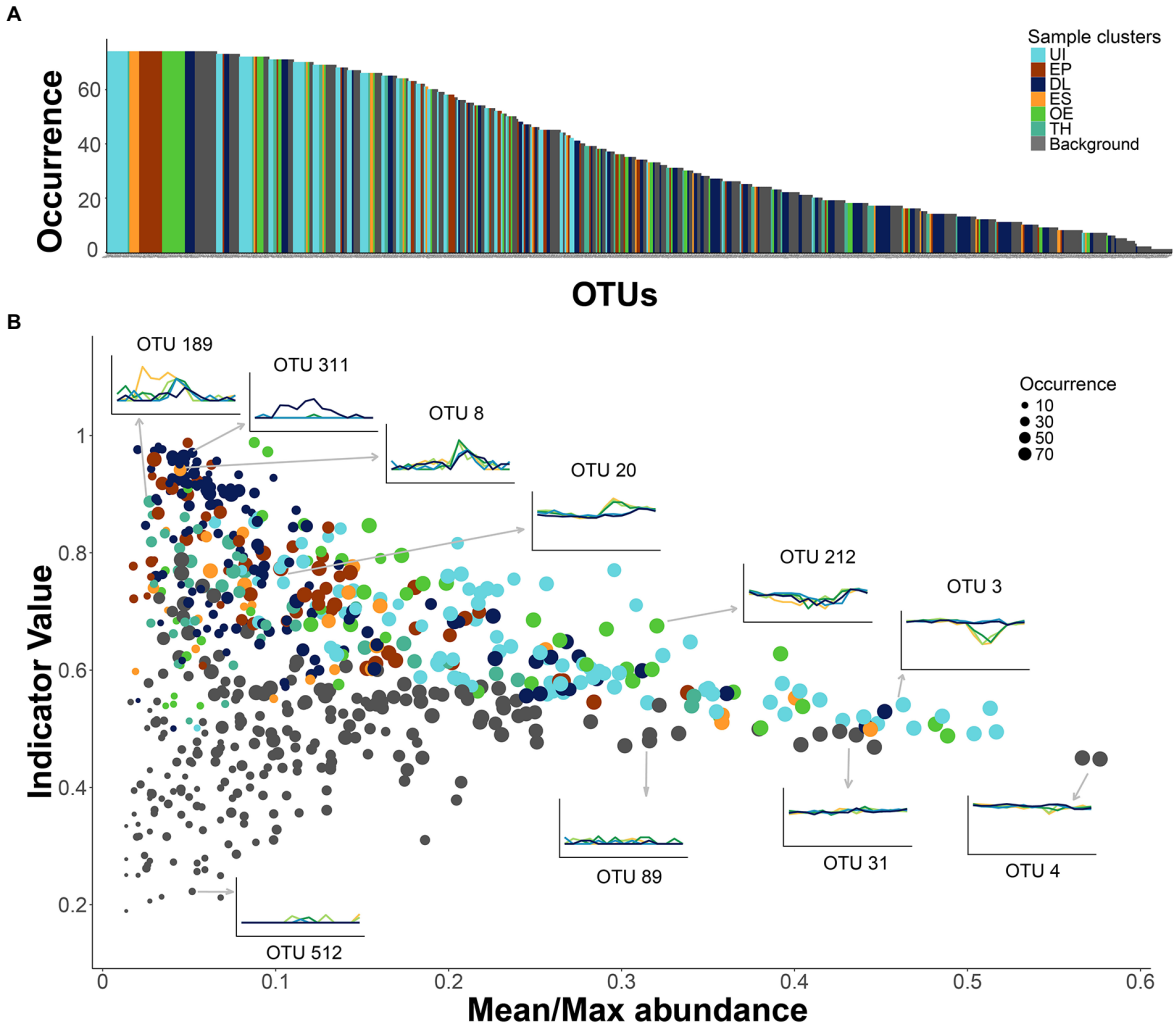
Patterns of occurrence and seasonality in the bacterioplankton community. **(A)** OTUs ranked by occurrence in the samples. Colors specify cluster indicators: UI (under-ice), TH (thaw and hypolimnion), ES (early stratification), EP (epilimnion), OE (overturn and early under-ice), and DL (deep layer). **(B)** Comparison of the OTU indicator values with the OTU’s mean and maximum abundance ratio in the samples. A larger ratio indicates flatter seasonal OTU profiles, that is, lower occasional blooming. The symbol size is proportional to OTU occurrence. The inserted plots show examples of the OTU abundance’s time series at each sampling depth (line colors as in Figure 2). Arrows indicate the corresponding OTU in the larger plot.

Some OTUs that were non-significant indicators of any seasonal cluster were as frequent in the samples as those with indicator significance (Figure 6A). Their relative sample abundance ranged from 8 to 32%. The lack of cluster significance was due to their stability throughout the year and across depths (Figure 6B). Some exceptions were rare OTUs, that appeared sporadically in a few samples (e.g., OTU-512; Figure 6B). Many OTUs affiliated with Actinobacteria showed stable gene densities (~50%, Supplementary Table S3), especially the family Sporichthyaceae and the hgcI-clade. The stability and abundance of two of them was particularly remarkable, OTU-4 (Figure 6B) and OTU-41. The stable OTUs constitute a background assemblage across all seasons (Supplementary Figure S2B) and a helpful benchmark for comparing the patterns of the indicator OTUs (Figure 6B). About one-third of the indicative OTUs were also present in many samples (>80%). The UI indicators were particularly ubiquitous across seasons and depths (Figure 6A), although there were also widespread indicators of other seasonal clusters. An OTU’s indicator value was achieved in two different ways. The UI and OE clusters’ indicators displayed a rather stable gene density in most samples and declined under specific conditions. For example, OTU-3 (Acidimicrobiia, CL500-29 marine group) and OTU-212 (Actinobacteria, PeM15) showed similar abundances over the year but declined in the ice-free epilimnion. By contrast, the indicator values of OTUs in the ES, EP, and TH clusters were related to their blooming during the respective seasonal periods; for example, OTU-20 (Gammaproteobacteria*, Limnohabitans*), EP cluster indicator; OTU-8 (Bacteroidia*, Flavobacterium*) ES indicator; and OTU-189 (Gammaproteobacteria, Oxalobacteraceae), TH indicator (Figure 6B). The indicator OTUs associated with the DL cluster also showed blooming patterns, but the main reason was their spatial segregation in the 60-m layer (e.g., OTU-311, Bacteroidia, BSV13; Figure 6B).

## Discussion

Our study indicates a relatively stable bacterioplankton community with a large core of permanently coexisting OTUs. About two-thirds of taxa were present in many samples with seasonally oscillating abundance, and some others showed stable abundances across time and space. This developed core community could be due to Lake Redon’s average renewal time of 4 years, with deep layers that may exceed a decade of water residence (Catalan, 1988), the small catchment compared to the lake area, and the limited stream network. Consequently, the proportion of randomly appearing OTUs does not agree with lakes located in complex networks of aquatic ecosystems, such as boreal lakes (Niño-García et al., 2017) or mountain lakes below the tree line (Vincent et al., 2022). Our findings also contrast the idea of a succession of communities that replace each other across seasons (Fuhrman et al., 2006; Crump et al., 2009; Eiler et al., 2012). Instead, the environmental oscillations seem to foster coexistence similar to what has been found for protists (Zufiaurre et al., 2021). Three main types of community members concerning seasonal patterns were found: (i) OTUs with stable abundance, non-sensitive to seasonal environmental fluctuations and depth gradients; (ii) OTUs with high abundance under ice and in the hypolimnion, declining only in the summer epilimnion, and (iii) OTUs with a low abundance that increased in the epilimnion when many other OTUs declined. The three types could be termed the resistant, ice-on-resilient, and ice-off-resilient components of the bacterioplankton core community.

### The resistant component

The large fraction of the bacterial community not showing significant seasonal variation included some of the most abundant and frequent OTUs. This stability suggests stress-tolerant organisms (Allison and Martiny, 2008). Most of them were assigned to the actinobacterial Sporichthyaceae family and hgcl-clade, which shows many features that can be beneficial in oligotrophic mountain lakes. Actinobacteria are ubiquitous in terrestrial and aquatic ecosystems (Monard et al., 2016), and the hgcl-clade is highly relevant in freshwaters (Newton et al., 2011). Actinobacteria show lower growth rates than many other phyla and have the capacity to store polyphosphates (Forbes et al., 2009) and process nitrogen-rich compounds (Ghylin et al., 2014). Furthermore, pigment production, strong cell wall, and DNA repair capacity protect Actinobacteria against high UV radiation (Warnecke et al., 2005). Besides, the hgcl-clade is endowed with actinorhodopsin (Sharma et al., 2009), which allows using sunlight energy for heterotrophic growth (Dwulit-Smith et al., 2018), and carotenoids that protect from high-radiation and oxidative stress. Their cell wall composition (Gram-positive) and small cell size were proposed to protect against grazing (Pernthaler, 2005), and, indeed, they are rarely predated by phagotrophic flagellates in mountain lakes (Ballen-Segura et al., 2017). Actinobacteria are not only of minute cell size but also among the most streamlined free-living microbes, with extremely small genome sizes (1.2–1.4 Mbp) reflecting evolutionary adaptation to the competition for limiting resources under oligotrophic conditions (Giovannoni et al., 2014). Recent genome analysis revealed a high degree of micro-diversification in some hgcl lineages (Neuenschwander et al., 2018; Okazaki et al., 2021) that might explain their global success in highly dynamic freshwater environments and, in our case, could explain resistance to seasonal changes.

### The ice-on-resilient component

The under-ice and transition seasonal clusters showed the highest OTU diversity, which agrees with observations from other lakes and marine ecosystems (Grzymski et al., 2012; Bertilsson et al., 2013; Christner et al., 2014). However, in the present study, many of the indicator taxa of ice-covered seasonal clusters were found across all seasons and depths. Indeed, their seasonal pattern resulted from a critical summer period in the upper layers for these taxa (0–20-m depth; e.g., OTU-3, Figure 6). They maintained their abundance in the hypolimnion and quickly recovered throughout the water column during the autumn mixing and early ice-covered period. When the harsh summer conditions in the upper layers decline, this capacity for recovery indicates a highly resilient community (Allison and Martiny, 2008; Shabarova et al., 2021) associated with low irradiance periods or layers (i.e., clusters UI, TH, and OE). Verrucomicrobiota families were mainly associated with the ice-covered period (UI, OE) and were sparse in summer samples. This phylum has been linked to winter (Aguilar and Sommaruga, 2020; Cruaud et al., 2020) and oligotrophic conditions (Kolmonen et al., 2011). More specifically, Verrucomicrobiaceae and Chthoniobacteraceae, related to the degradation of organic matter under ice (Tran et al., 2018) and extracellular polymeric substances (Bohórquez et al., 2017), were abundant after autumn overturn and the beginning of ice cover, the period following the last seasonal phytoplankton growth peaks (Felip and Catalan, 2000). Other indicators of the ice-covered period were some Acidobacteriota, which have also been associated with winter conditions in the Arctic tundra (Männistö et al., 2013). Acidimicrobiia related to the CL500-29-marine group also showed a winter resilience pattern. They have been found primarily in marine ecosystems and deep oligotrophic lakes (Zwart et al., 2002) and are considered generalists.

The bacterial composition of the deep layers (DL) included many under ice (UI) taxa but also many other OTUs not present in other samples, with functional traits different from the rest of the community (Supplementary Appendix S1). The basin shape of the lake changes at about 60-m depths, markedly increasing the sediment proportion in contact with the water column layers. The deepest lake volume (> 60–73-m depth) is deficient in oxygen and enriched in CO_2_, NH_4_^+^, NO_3_^−^, and SO_4_^2−^, among other compounds, thus supporting a large variety of metabolisms. In late winter, the 60-m layer is influenced by this volume with close sediment contact, resulting in a highly diverse ecotone community.

The thawing period and deep hypolimnion (cluster TH) favored Burkholderiales, an order of the Gammaproteobacteria formerly known as Betaproteobacteria, one of the most abundant and diverse lineages in freshwater ecosystems (Newton et al., 2011). They might have responded to the increase in dissolved inorganic nitrogen during thaw and spring mixing (Figure 4B), which agrees with previous studies (Salcher et al., 2008). This relationship was probably mediated by the phytoplankton growth, particularly cryptophytes and chlorophytes, and their extracellular production (Šimek et al., 2011). Both protist groups in Lake Redon are usually found in deep layers, forming a deep chlorophyll maximum in the upper hypolimnion (Felip et al., 1999a). The psychrotrophic genus *Polaromonas*, one of the most abundant taxa in glaciers (Gawor et al., 2016), was also particularly abundant at this point when the melting water of the ice cover mixed with the water column. Cluster TH fits in the transition between typical ice-on and ice-off conditions.

### The ice-off-resilient component

The summer epilimnetic conditions were unfavorable for many community members. Those that increase in abundance can be seen as a component of the core community that only takes advantage in extreme situations for the rest of the bacterioplankton (Pernthaler, 2017). These organisms should be able to increase in high UV irradiance, temperatures well above the typical values found most of the year, and organic matter of poor quality (e.g., high C:P and N:P ratios). Although the OTU richness was low in these conditions, the 16S rRNA gene abundance did not differ from the under-ice values at 2, 10, and 20-m depths, suggesting that the growth of ice-free-resilient taxa compensated for the decline of the others, although gene copies do not necessarily reflect population dynamics. Some Bacteroidota were among the best indicators of the epilimnion community (ES and EP clusters). This phylum includes organisms highly resistant to UV radiation (Alonso-Sáez et al., 2006) and can also grow on media enriched in carbon and low in nutrients (Fierer et al., 2007). Although UV resistance appears mandatory, other factors may influence the Bacteroidota groups present (Spirosomaceae, Chitinophagaceae, and Flavobacteriaceae), such as resistance to grazing and preference for higher temperatures (Pernthaler, 2005; Neuenschwander et al., 2015). The highest presence of Gammaproteobacteria in the epilimnion corresponded to *Limnohabitans* and *Rhodoferax,* two aerobic anoxygenic phototrophic bacteria (AAPs) typically associated with productive periods (Šimek et al., 2013). However, epilimnetic waters in Lake Redon are much less productive than deeper waters and overturn periods (Felip and Catalan, 2000). Therefore, AAPs relevance should be related to other factors such as grazing or irradiance not clearly identified yet (Ruiz-González et al., 2020).

### Perspective on a shifting climate

Our study indicates that under-ice conditions may fuel high-mountain bacterioplankton diversity in deep lakes covered by ice for about half the year. Therefore, it could be speculated that shorter ice-cover periods, an ongoing global trend (Sharma et al., 2019), could jeopardize the current bacterioplankton diversity. Nevertheless, the study also shows a high resilience of the under-ice assemblages if refugia exist, such as a deep hypolimnion. Many under-ice taxa also persisted during mixing periods, and only epilimnetic conditions appeared more harmful. The answer to expected changes may be the difference between the under-ice (UI) and autumn overturn-early ice-cover (OE) seasonal clusters. The two seasonal clusters show compositional features in common, but OE has lower diversity. Delayed ice formation, or eventual ice-cover loss, will result in extended winter complete-mixing periods. Accordingly, we could expect a decline in Verrucomicrobia and Acidimicrobiia high-rank taxonomic diversity, which show their diversity predominantly during the under-ice period. Bacteroidia and Gammaproteobacteria could be favored as they are characteristic of the OE period but do not show a similar richness as the under-ice assemblages. Our findings show that shorter ice-on periods can result in an overall decline in bacterioplankton diversity. Nevertheless, provided the resilience of the under-ice assemblages during mixing periods and the hypolimnion partial refuge, the ice cover reduction must be extreme and persistent, far beyond the one-month fluctuations observed during the last decades (Camarero and Catalan, 2012). We can speculate that a non-linear response could be expected, with a tipping point at some relatively short ice-cover duration.

## Data availability statement

The data presented in the study are deposited in: https://www.ncbi.nlm.nih.gov/genbank/, with accession numbers MZ245731-MZ246381.

## Author contributions

JC, MF, and LC planned the study. JC, SH, and LC contributed to reagents, materials, and analysis tools. LC and MS-F performed fieldwork and chemical analyses. AZ, JJ, GB-R, and SH performed molecular analyses. AZ, MF, and JC performed numerical analyses and wrote the first draft of the manuscript. All authors contributed to the article and approved the submitted version.

## Funding

The research was funded by grants from the Spanish Government, Ministerio de Ciencia e Innovación, NitroPir (CGL2010–19737), and Transfer (CGL2016–80124-C2-1-P) and the Catalan Government GECA (2017 SGR 910).

## Conflict of interest

The authors declare that the research was conducted in the absence of any commercial or financial relationships that could be construed as a potential conflict of interest.

## Publisher’s note

All claims expressed in this article are solely those of the authors and do not necessarily represent those of their affiliated organizations, or those of the publisher, the editors and the reviewers. Any product that may be evaluated in this article, or claim that may be made by its manufacturer, is not guaranteed or endorsed by the publisher.

## Footnotes

^1^http://www.bioinformatics.babraham.ac.uk/projects/fastqc/ (Accessed August 10, 2022).

^2^http://hannonlab.cshl.edu/fastx_toolkit/index.html (Accessed August 10, 2022).
