# Supplementary material for: Bacterioplankton seasonality in deep high-mountain lakes
Source: Front Microbiol. 2022 Sep 14;13:935378. doi: 10.3389/fmicb.2022.935378 (PMC9519062; doi:10.3389/fmicb.2022.935378)
Supplement: Supplementary file 1 [file Data_Sheet_1.docx]

Supplementary Material

**CONTENT PAGE**

**Figure S1** Exponential Shannon entropy variation in seasonal clusters 1

**Figure S2** Relative gene abundances of the bacterial families in clusters 2

**Appendix S1**Functional diversity in deep layers (DL cluster) 3

**Table S1** Primers and details for the 16S rRNA PCR and Illumina sequencing 5

**Table S2** Environmental variables summary statistics 6

**Table S3** Number of OTUs by high-rank taxonomy of the indicator and background clusters 7

**Table S4** Taxonomic assignment of the OTUs. 8


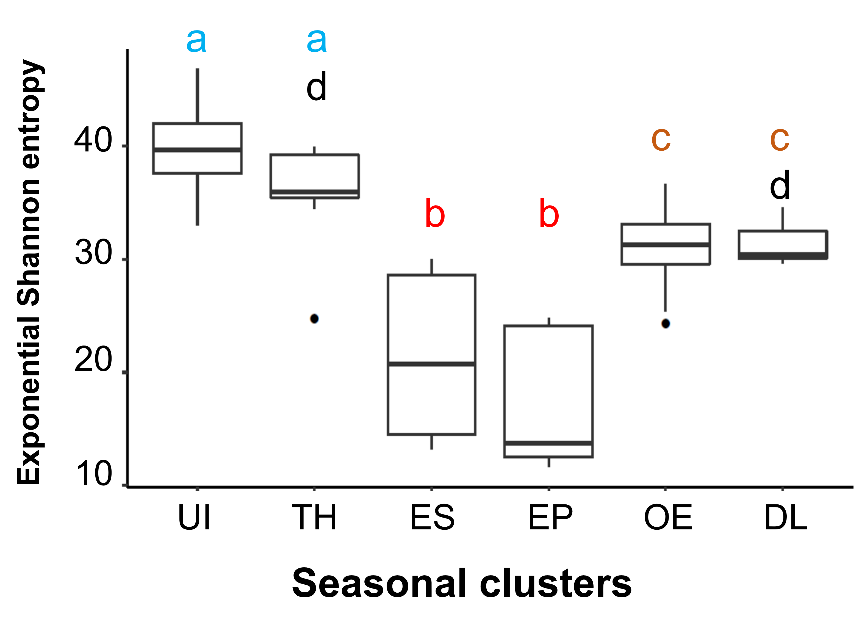


**Fig. S1.** Exponential Shannon entropy distributions as indicators of diversity variation among seasonal clusters: UI, under-ice; TH, thaw, and hypolimnion; ES, early stratification, EP, epilimnion, OE, overturn and early under-ice; DL, deep layers. ANOVA indicated overall significant differences (P<0.001), and post hoc pairwise comparisons with the HSD Tukey test are indicated with letters on top of the boxplots. The same letter indicates no significant difference (P>0.05).

**
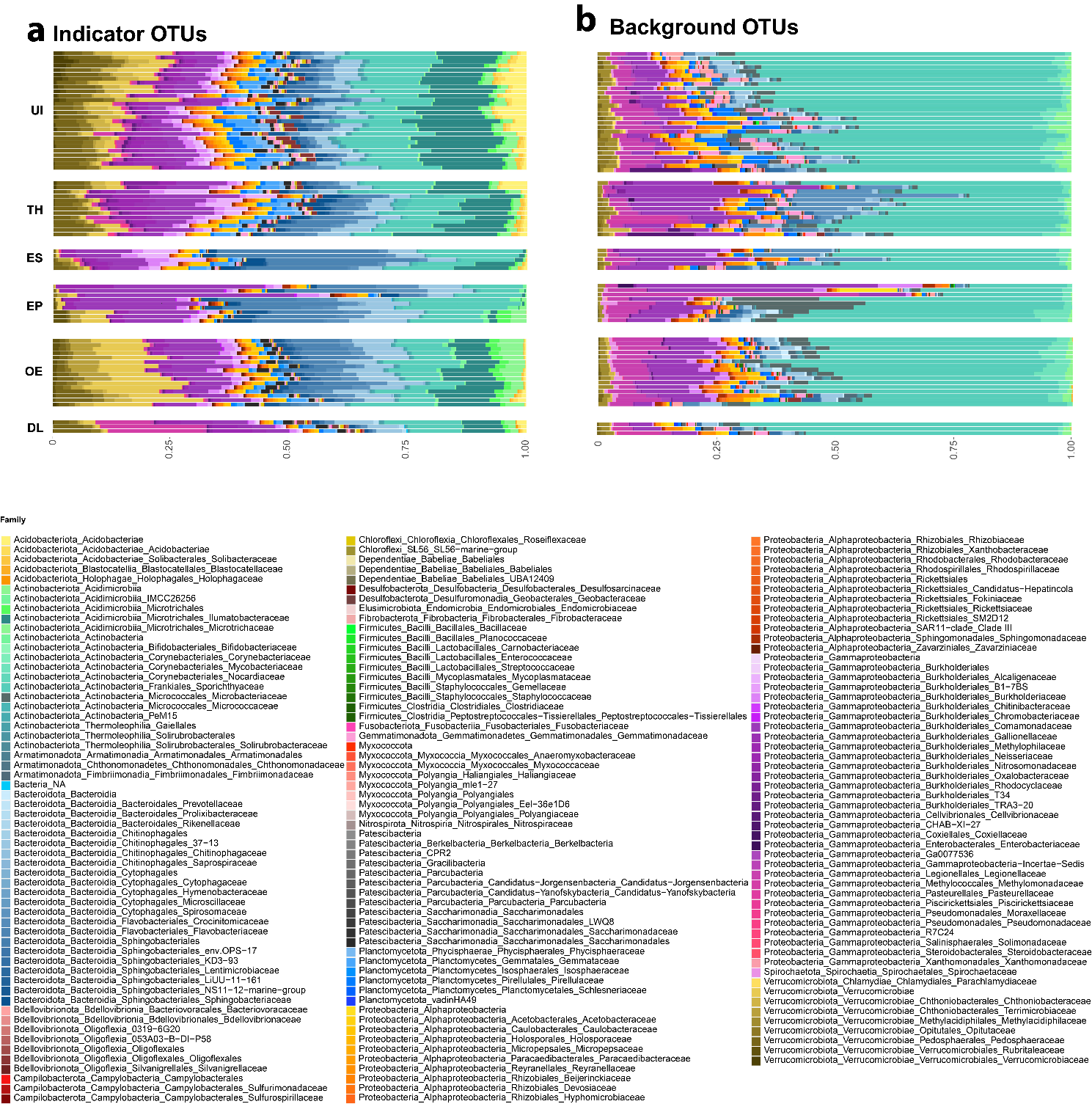
**

**Fig. S2.** Relative abundance of bacterial families in the samples differentiating between indicator (a) and background OTUs (b). Samples are sorted by date and depth within cluster groups. UI, under-ice; TH, thaw, and hypolimnion; ES, early stratification, EP, epilimnion, OE, overturn and early under-ice; DL, deep layers. NA, not assigned.

# Appendix S1. Functional diversity in deep layers (DL cluster)

Among the DL cluster indicator OTUs, we found *Geobacter* with the capacity for CO oxidation [1]; the methanotrophs *Crenothrix and Methylobacter*, [2-4]; the methylotrophs Methylophilaceae Methylotenera [2,5] and Rhodocyclacea [6]; nitrifiers as the commamox *Nitrospira* [7]; denitrifiers as *Crenothrix* [8], dissimilatory nitrate reducers to ammonium, some Bacteroidales [1]; organisms able of nitrogen fixation such as *Endomicrobium* [9]; sulfur oxidation OTUs as *Sulfurimonas* [10,11]; sulfate reducers, *Desulfatirhabdium* [12], and *Geobacter* [13,14]; Fe (III) reducers as *Geobacter* and *Geothrix* [15] and Hg methylators as Desulfobacterales, *Geobacter* and Myxococcaceae [13,16,17]. OTUs with reduced genome size, cell size, and metabolic capacity were also prevalent in the DL cluster, as several types of Patescisbacteria (CPR2, Parcubacteria, and Saccharimonadales) [18] and Rickettsiales [19]

**Literature cited**

1. Llorens-Marès, T.; Yooseph, S.; Goll, J.; Hoffman, J.; Vila-Costa, M.; Borrego, C.M.; Dupont, C.L.; Casamayor, E.O. Connecting biodiversity and potential functional role in modern euxinic environments by microbial metagenomics. *The ISME journal* **2015**, *9*, 1648.

2. DeLong, E.F.; Lory, S.; Stackebrandt, E.; Thompson, F.L. *The Prokaryotes: Alphaproteobacteria and Betaproteobacteria*; Springer-Verlag Berlin Heidelberg, 2014.

3. Knief, C. Diversity and habitat preferences of cultivated and uncultivated aerobic methanotrophic bacteria evaluated based on pmoA as molecular marker. *Frontiers in microbiology* **2015**, *6*, 1346.

4. Samad, M.S.; Bertilsson, S. Seasonal variation in abundance and diversity of bacterial methanotrophs in five temperate lakes. *Frontiers in microbiology* **2017**, *8*, 142.

5. Ricão Canelhas, M.; Denfeld, B.A.; Weyhenmeyer, G.A.; Bastviken, D.; Bertilsson, S. Methane oxidation at the water‐ice interface of an ice‐covered lake. *Limnol Oceanogr* **2016**, *61*.

6. Smalley, N.E.; Taipale, S.; De Marco, P.; Doronina, N.V.; Kyrpides, N.; Shapiro, N.; Woyke, T.; Kalyuzhnaya, M.G. Functional and genomic diversity of methylotrophic Rhodocyclaceae: description of *Methyloversatilis discipulorum* sp. nov. *Int J Syst Evol Microbiol* **2015**, *65*, 2227-2233.

7. Daims, H.; Lebedeva, E.V.; Pjevac, P.; Han, P.; Herbold, C.; Albertsen, M.; Jehmlich, N.; Palatinszky, M.; Vierheilig, J.; Bulaev, A., et al. Complete nitrification by *Nitrospira* bacteria. *Nature* **2015**, *528*, 504, doi:10.1038/nature16461.

8. Oswald, K.; Graf, J.S.; Littmann, S.; Tienken, D.; Brand, A.; Wehrli, B.; Albertsen, M.; Daims, H.; Wagner, M.; Kuypers, M.M.M., et al. *Crenothrix* are major methane consumers in stratified lakes. *The Isme Journal* **2017**, *11*, 2124, doi:10.1038/ismej.2017.77.

9. Zheng, H.; Dietrich, C.; Radek, R.; Brune, A. *Endomicrobium proavitum*, the first isolate of Endomicrobia class. nov. (phylum Elusimicrobia) – an ultramicrobacterium with an unusual cell cycle that fixes nitrogen with a Group IV nitrogenase. *Environ Microbiol* **2016**, *18*, 191-204.

10. Han, C.; Kotsyurbenko, O.; Chertkov, O.; Held, B.; Lapidus, A.; Nolan, M.; Lucas, S.; Hammon, N.; Deshpande, S.; Cheng, J.-F. Complete genome sequence of the sulfur compounds oxidizing chemolithoautotroph *Sulfuricurvum kujiense* type strain (YK-1 T). *Standards in genomic sciences* **2012**, *6*, 94.

11. Kodama, Y.; Watanabe, K. *Sulfuricurvum kujiense* gen. nov., sp. nov., a facultatively anaerobic, chemolithoautotrophic, sulfur-oxidizing bacterium isolated from an underground crude-oil storage cavity. *Int J Syst Evol Microbiol* **2004**, *54*, 2297-2300.

12. Almstrand, R.; Pinto, A.J.; Figueroa, L.A.; Sharp, J.O. Draft genome sequence of a novel Desulfobacteraceae member from a sulfate-reducing bioreactor metagenome. *Genome announcements* **2016**, *4*, e01540-01515.

13. Bravo, A.G.; Zopfi, J.; Buck, M.; Xu, J.; Bertilsson, S.; Schaefer, J.K.; Poté, J.; Cosio, C. Geobacteraceae are important members of mercury-methylating microbial communities of sediments impacted by waste water releases. *The ISME journal* **2018**, *12*, 802.

14. Fleming, E.J.; Mack, E.E.; Green, P.G.; Nelson, D.C. Mercury methylation from unexpected sources: molybdate-inhibited freshwater sediments and an iron-reducing bacterium. *Appl Environ Microbiol* **2006**, *72*, 457-464.

15. Lovley, D.R.; Ueki, T.; Zhang, T.; Malvankar, N.S.; Shrestha, P.M.; Flanagan, K.A.; Aklujkar, M.; Butler, J.E.; Giloteaux, L.; Rotaru, A.-E. Geobacter: the microbe electric's physiology, ecology, and practical applications. In *Adv Microb Physiol*, Elsevier: 2011; Vol. 59, pp. 1-100.

16. Parks, J.M.; Johs, A.; Podar, M.; Bridou, R.; Hurt, R.A.; Smith, S.D.; Tomanicek, S.J.; Qian, Y.; Brown, S.D.; Brandt, C.C. The genetic basis for bacterial mercury methylation. *Science* **2013**, *339*, 1332-1335.

17. Gilmour, C.C.; Podar, M.; Bullock, A.L.; Graham, A.M.; Brown, S.D.; Somenahally, A.C.; Johs, A.; Hurt Jr, R.A.; Bailey, K.L.; Elias, D.A. Mercury methylation by novel microorganisms from new environments. *Environ Sci Technol* **2013**, *47*, 11810-11820.

18. Maria-Cecilia, C.; Paul-Adrian, B.; Adrian-Stefan, A.; Yusuke, O.; Shin-ichi, N.; Markus, H.; Vinicius Silva, K.; Paul, L.; Rohit, G.; Michaela, M.S. Ecogenomics sheds light on diverse lifestyle strategies in freshwater CPR. *Research Square* **2022**, *pre-print*, doi:10.21203/rs.3.rs-776685/v2.

19. McCutcheon, J.P.; Moran, N.A. Extreme genome reduction in symbiotic bacteria. *Nature Reviews Microbiology* **2012**, *10*, 13.

Table S1**.** Primers and thermal cycling details for quantification (quantitative real-time PCR) and sequencing (Illumina) of the 16S rRNA gene.

| **Target** | **Gene** | **Sequence** | **Thermal cycling** |  | **Reference** |
| --- | --- | --- | --- | --- | --- |
|  | Primer | **(5'-3')** |  |  |  |
| Bacteria 16S rRNA | **16S rRNA** |  | (95°C, 7 min) x1 |  | López-Gutierrez *et al.,* 2004 |
|  | 341F | CCT ACG GGA GGC AGC AG | (95°C, 15 s; 60°C 30 s; 72°C, 30 s; 80°C, 10 s) x 35 | |  |
|  | 534R | ATT ACC GCG GCT GCT GGC A | (95°C, 15 s; 60 to 95°C, 10s increment 0.5°) x 1 |  |  |
|  |  |  |  |  |  |
| Prokaryote 16S rRNA | **16S rRNA** |  | (98°C, 3 min) x 1 |  | Takahashi *et al.,* 2014 |
|  | Pro341F | TCGTCGGCAGCGTCAGATGTGTATAAGAGACAGNNNNCCTACGGGNBGCASCAG | (98°C, 30s; 55°C, 30s; 72°C, 45s) x 25 |  |  |
|  | Pro805R | GTCTCGTGGGCTCGGAGATGTGTATAAGAGACAGNNNNGACTACNVGGGTATCTAATCC | (72°C, 5 min) x 1 |  |  |

Table S2**.** Summary of the environmental variables (74 samples).

| Environmental variable | Units | Min | Max | Median | Mean | Standard deviation |
| --- | --- | --- | --- | --- | --- | --- |
| Iz |  | 0 | 0.92 | 0 | 0.17 | 0.29 |
| Temp | ^o^C | 0.7 | 13.2 | 3.1 | 3.8 | 2.7 |
| O_2_ | mg L^-1^ | 0.9 | 11.0 | 9.4 | 8.8 | 2.1 |
| Chla | µg L^-1^ | 0.01 | 1.5 | 0.29 | 0.42 | 0.38 |
| Chla:PC | w/w | 0.0002 | 0.0118 | 0.0032 | 0.0037 | 0.0025 |
| PC | µmol L^-1^ | 0.99 | 26.8 | 9.2 | 9.0 | 4.9 |
| PN | µmol L^-1^ | 0.31 | 1.95 | 1.12 | 1.01 | 0.39 |
| PP | µmol L^-1^ | 0.02 | 0.08 | 0.05 | 0.05 | 0.01 |
| C:N (seston) | a/a | 2.1 | 18.4 | 8.5 | 8.6 | 2.94 |
| C:P (seston) | a/a | 24 | 497 | 160 | 180 | 105 |
| N:P (seston) | a/a | 8.2 | 48.5 | 19.0 | 20.4 | 9.2 |
| DOC | mg L^-1^ | 0.1 | 0.5 | 0.3 | 0.3 | 0.1 |
| pH |  | 5.94 | 6.95 | 6.56 | 6.53 | 0.23 |
| CO_2_ | µmol L^-1^ | 3.6 | 150 | 26 | 36 | 31 |
| NH_4_^+^ | µmol L^-1^ | 0.2 | 10.2 | 1.2 | 1.7 | 1.5 |
| NO_2_^-^ | µmol L^-1^ | 0.00 | 0.09 | 0.03 | 0.03 | 0.02 |
| NO_3_^-^ | µmol L^-1^ | 3 | 13 | 4 | 5 | 2 |
| DON | µmol L^-1^ | 0.3 | 10 | 3 | 3 | 2 |
| SRP | µmol L^-1^ | 0.003 | 0.054 | 0.010 | 0.011 | 0.008 |
| DOP | µmol L^-1^ | 0.000 | 0.078 | 0.009 | 0.010 | 0.009 |
| SO_4_^2-^ | µmol L^-1^ | 6 | 10.5 | 7.5 | 7.5 | 0.5 |
| DRSi | µmol L^-1^ | 8 | 23 | 9 | 10 | 3 |

**Table S3.** Number of OTUs by high-rank taxonomy of the indicator and background clusters

| **Phylum** | **Class** | **UI** | **TH** | **ES** | **EP** | **OE** | **DL** | **BG** | **Total** |
| --- | --- | --- | --- | --- | --- | --- | --- | --- | --- |
| Acidobacteriota | Acidobacteriae | 3 | 1 | 0 | 0 | 0 | 0 | 0 | 4 |
| Acidobacteriota | Blastocatellia | 0 | 0 | 0 | 0 | 1 | 0 | 1 | 2 |
| Acidobacteriota | Holophagae | 0 | 0 | 0 | 0 | 0 | 3 | 0 | 3 |
| Actinobacteriota | Acidimicrobiia | 9 | 0 | 0 | 0 | 5 | 3 | 3 | 20 |
| Actinobacteriota | Actinobacteria | 6 | 1 | 2 | 8 | 6 | 6 | 27 | 56 |
| Actinobacteriota | Thermoleophilia | 2 | 0 | 0 | 1 | 0 | 1 | 4 | 8 |
| Armatimonadota | Armatimonadia | 0 | 0 | 0 | 0 | 0 | 0 | 1 | 1 |
| Armatimonadota | Chthonomonadetes | 0 | 0 | 0 | 0 | 0 | 1 | 0 | 1 |
| Armatimonadota | Fimbriimonadia | 0 | 0 | 0 | 0 | 0 | 0 | 1 | 1 |
| Bacteroidota | Bacteroidia | 8 | 7 | 11 | 20 | 12 | 29 | 34 | 121 |
| Bdellovibrionota | Bdellovibrionia | 2 | 0 | 0 | 0 | 0 | 1 | 4 | 7 |
| Bdellovibrionota | Oligoflexia | 2 | 2 | 0 | 2 | 0 | 2 | 9 | 17 |
| Campilobacterota | Campylobacteria | 0 | 0 | 0 | 0 | 0 | 3 | 1 | 4 |
| Chloroflexi | Chloroflexia | 0 | 0 | 0 | 0 | 0 | 1 | 0 | 1 |
| Chloroflexi | SL56 | 1 | 0 | 0 | 0 | 0 | 0 | 0 | 1 |
| Dependentiae | Babeliae | 1 | 0 | 0 | 0 | 1 | 3 | 1 | 6 |
| Desulfobacterota | Desulfobacteria | 0 | 0 | 0 | 0 | 0 | 1 | 0 | 1 |
| Desulfobacterota | Desulfuromonadia | 0 | 0 | 0 | 0 | 0 | 4 | 0 | 4 |
| Elusimicrobiota | Endomicrobia | 0 | 0 | 0 | 0 | 0 | 1 | 0 | 1 |
| Fibrobacterota | Fibrobacteria | 0 | 0 | 0 | 0 | 0 | 0 | 1 | 1 |
| Firmicutes | Bacilli | 0 | 0 | 0 | 0 | 2 | 1 | 9 | 12 |
| Firmicutes | Clostridia | 0 | 0 | 0 | 0 | 0 | 0 | 6 | 6 |
| Fusobacteriota | Fusobacteriia | 0 | 0 | 0 | 0 | 0 | 3 | 0 | 3 |
| Gemmatimonadota | Gemmatimonadetes | 0 | 1 | 0 | 0 | 0 | 0 | 1 | 2 |
| Myxococcota | Myxococcia | 0 | 0 | 0 | 0 | 0 | 2 | 0 | 2 |
| Myxococcota | Polyangia | 3 | 0 | 0 | 0 | 0 | 0 | 3 | 6 |
| Nitrospirota | Nitrospiria | 0 | 0 | 0 | 0 | 0 | 1 | 0 | 1 |
| Patescibacteria | Berkelbacteria | 0 | 0 | 0 | 0 | 0 | 1 | 0 | 1 |
| Patescibacteria | CPR2 | 0 | 0 | 0 | 0 | 0 | 1 | 0 | 1 |
| Patescibacteria | Gracilibacteria | 0 | 0 | 0 | 1 | 0 | 0 | 0 | 1 |
| Patescibacteria | Parcubacteria | 0 | 0 | 0 | 0 | 0 | 8 | 1 | 9 |
| Patescibacteria | Saccharimonadia | 0 | 1 | 0 | 0 | 2 | 7 | 3 | 13 |
| Planctomycetota | Phycisphaerae | 2 | 0 | 0 | 0 | 0 | 0 | 0 | 2 |
| Planctomycetota | Planctomycetes | 5 | 0 | 1 | 0 | 1 | 2 | 5 | 14 |
| Planctomycetota | vadinHA49 | 0 | 0 | 0 | 0 | 0 | 0 | 1 | 1 |
| Proteobacteria | Alphaproteobacteria | 15 | 4 | 8 | 8 | 6 | 13 | 55 | 109 |
| Proteobacteria | Gammaproteobacteria | 18 | 24 | 5 | 16 | 9 | 32 | 57 | 161 |
| Spirochaetota | Spirochaetia | 0 | 0 | 0 | 0 | 0 | 0 | 1 | 1 |
| Verrucomicrobiota | Chlamydiae | 0 | 0 | 0 | 0 | 0 | 0 | 2 | 2 |
| Verrucomicrobiota | Verrucomicrobiae | 14 | 2 | 1 | 1 | 5 | 7 | 9 | 39 |
| Bacteria-NA | NA | 0 | 0 | 0 | 1 | 0 | 3 | 1 | 5 |
| Total OTUs |  | 91 | 43 | 28 | 58 | 50 | 140 | 241 |  |
| Total Classes |  | 15 | 9 | 6 | 8 | 11 | 26 | 25 |  |
| UI, under-ice; TH, thaw and hypolimnion; ES, early stratification, EP, epilimnion, OE, overturn and early under-ice; DL, deep layers; BG, background assemblage without indicator relevance. NA, not assigned. | | | | | | | | | |

Table S4**.** Taxonomic assignment of OTUs.

| **OTU ID** | **Phylum** | **Class** | **Order** | **Family** | **Genus** | **Cluster** |
| --- | --- | --- | --- | --- | --- | --- |
| 1 | Proteobacteria | Gammaproteobacteria | Burkholderiales | Comamonadaceae | Polaromonas | TH |
| 2 | Bacteroidota | Bacteroidia | Flavobacteriales | Flavobacteriaceae | Flavobacterium | ES |
| 3 | Actinobacteriota | Acidimicrobiia | Microtrichales | Ilumatobacteraceae | CL500-29-marine-group | UI |
| 4 | Actinobacteriota | Actinobacteria | Frankiales | Sporichthyaceae | hgcI-clade | BG |
| 5 | Proteobacteria | Gammaproteobacteria | Burkholderiales | Comamonadaceae | Polaromonas | BG |
| 6 | Actinobacteriota | Actinobacteria | Frankiales | Sporichthyaceae | hgcI-clade | EP |
| 7 | Verrucomicrobiota | Verrucomicrobiae |  |  |  | OE |
| 8 | Bacteroidota | Bacteroidia | Flavobacteriales | Flavobacteriaceae | Flavobacterium | ES |
| 9 | Actinobacteriota | Acidimicrobiia | Microtrichales | Ilumatobacteraceae | CL500-29-marine-group | UI |
| 10 | Bacteroidota | Bacteroidia | Cytophagales | Spirosomaceae | Pseudarcicella | EP |
| 11 | Proteobacteria | Gammaproteobacteria | Burkholderiales | Methylophilaceae | Methylotenera | TH |
| 13 | Bacteroidota | Bacteroidia | Chitinophagales | Chitinophagaceae | Sediminibacterium | EP |
| 14 | Proteobacteria | Gammaproteobacteria | Burkholderiales | Comamonadaceae | Rhodoferax | EP |
| 15 | Actinobacteriota | Actinobacteria | Frankiales | Sporichthyaceae | Sporichthyaceae | EP |
| 16 | Proteobacteria | Alphaproteobacteria | Caulobacterales | Caulobacteraceae | Caulobacter | ES |
| 17 | Planctomycetota | Planctomycetes | Gemmatales | Gemmataceae |  | UI |
| 18 | Actinobacteriota | Actinobacteria | Frankiales | Sporichthyaceae | hgcI-clade | ES |
| 19 | Proteobacteria | Gammaproteobacteria | Burkholderiales | Comamonadaceae | Limnohabitans | EP |
| 20 | Proteobacteria | Gammaproteobacteria | Burkholderiales | Comamonadaceae | Limnohabitans | EP |
| 21 | Acidobacteriota | Acidobacteriae |  |  |  | UI |
| 22 | Proteobacteria | Gammaproteobacteria | Burkholderiales | Burkholderiaceae | Polynucleobacter | ES |
| 23 | Proteobacteria | Gammaproteobacteria | Burkholderiales | Alcaligenaceae | GKS98-freshwater-group | ES |
| 24 | Verrucomicrobiota | Verrucomicrobiae |  |  |  | OE |
| 25 | Proteobacteria | Gammaproteobacteria | Burkholderiales | Methylophilaceae | Candidatus-Methylopumilus | UI |
| 26 | Bacteroidota | Bacteroidia | Chitinophagales | Chitinophagaceae | Parasediminibacterium | ES |
| 27 | Verrucomicrobiota | Verrucomicrobiae | Chthoniobacterales | Terrimicrobiaceae | FukuN18-freshwater-group | OE |
| 29 | Bacteroidota | Bacteroidia | Sphingobacteriales | Sphingobacteriaceae | Pedobacter | ES |
| 30 | Bacteroidota | Bacteroidia | Chitinophagales | Chitinophagaceae | Ferruginibacter | ES |
| 31 | Actinobacteriota | Actinobacteria | Frankiales | Sporichthyaceae | hgcI-clade | BG |
| 32 | Proteobacteria | Gammaproteobacteria | Burkholderiales | Alcaligenaceae | GKS98-freshwater-group | TH |
| 34 | Bacteroidota | Bacteroidia | Sphingobacteriales | NS11-12-marine-group | NS11-12-marine-group | EP |
| 35 | Verrucomicrobiota | Verrucomicrobiae | Pedosphaerales | Pedosphaeraceae | SH3-11 | DL |
| 36 | Proteobacteria | Gammaproteobacteria | Burkholderiales | Gallionellaceae | Candidatus-Nitrotoga | DL |
| 37 | Patescibacteria | Saccharimonadia | Saccharimonadales | Saccharimonadales | Saccharimonadales | DL |
| 38 | Actinobacteriota | Acidimicrobiia | Microtrichales | Ilumatobacteraceae | CL500-29-marine-group | UI |
| 39 | Verrucomicrobiota | Verrucomicrobiae | Pedosphaerales | Pedosphaeraceae |  | TH |
| 40 | Verrucomicrobiota | Verrucomicrobiae | Opitutales | Opitutaceae | Lacunisphaera | DL |
| 41 | Actinobacteriota | Actinobacteria | Frankiales | Sporichthyaceae |  | BG |
| 42 | Actinobacteriota | Acidimicrobiia |  |  |  | UI |
| 43 | Actinobacteriota | Acidimicrobiia |  |  |  | OE |
| 44 | Bacteroidota | Bacteroidia | Sphingobacteriales | env.OPS-17 | env.OPS-17 | OE |
| 45 | Bacteroidota | Bacteroidia | Sphingobacteriales | NS11-12-marine-group | NS11-12-marine-group | UI |
| 46 | Actinobacteriota | Actinobacteria | Frankiales | Sporichthyaceae | hgcI-clade | UI |
| 47 | Proteobacteria | Gammaproteobacteria | Legionellales | Legionellaceae | Legionella | BG |
| 48 | Verrucomicrobiota | Verrucomicrobiae | Chthoniobacterales | Chthoniobacteraceae | Chthoniobacter | UI |
| 50 | Proteobacteria | Gammaproteobacteria | Methylococcales | Methylomonadaceae | Methylobacter | DL |
| 51 | Bacteroidota | Bacteroidia | Chitinophagales | Chitinophagaceae | Sediminibacterium | OE |
| 52 | Actinobacteriota | Acidimicrobiia | Microtrichales | Ilumatobacteraceae | CL500-29-marine-group | OE |
| 53 | Actinobacteriota | Actinobacteria | Frankiales | Sporichthyaceae | hgcI-clade | ES |
| 54 | Verrucomicrobiota | Verrucomicrobiae | Pedosphaerales | Pedosphaeraceae |  | OE |
| 55 | Bacteroidota | Bacteroidia | Flavobacteriales | Flavobacteriaceae | Flavobacterium | EP |
| 56 | Actinobacteriota | Acidimicrobiia | Microtrichales |  |  | OE |
| 57 | Bacteroidota | Bacteroidia | Flavobacteriales | Flavobacteriaceae | Flavobacterium | EP |
| 58 | Proteobacteria | Gammaproteobacteria | Burkholderiales | Oxalobacteraceae |  | TH |
| 59 | Planctomycetota | Phycisphaerae | Phycisphaerales | Phycisphaeraceae | CL500-3 | UI |
| 60 | Proteobacteria | Alphaproteobacteria | Micropepsales | Micropepsaceae |  | UI |
| 61 | Planctomycetota | Planctomycetes | Pirellulales | Pirellulaceae |  | UI |
| 62 | Bacteroidota | Bacteroidia | Flavobacteriales | Flavobacteriaceae | Flavobacterium | BG |
| 63 | Proteobacteria | Gammaproteobacteria | Burkholderiales | Comamonadaceae | Limnohabitans | OE |
| 64 | Bacteroidota | Bacteroidia | Flavobacteriales | Flavobacteriaceae | Flavobacterium | EP |
| 65 | Proteobacteria | Gammaproteobacteria | Burkholderiales | Comamonadaceae | Paucibacter | BG |
| 66 | Bacteroidota | Bacteroidia | Cytophagales | Spirosomaceae | Arcicella | BG |
| 67 | Verrucomicrobiota | Verrucomicrobiae | Pedosphaerales | Pedosphaeraceae | SH3-11 | UI |
| 68 | Verrucomicrobiota | Verrucomicrobiae | Opitutales | Opitutaceae |  | UI |
| 69 | Verrucomicrobiota | Verrucomicrobiae |  |  |  | UI |
| 70 | Bdellovibrionota | Oligoflexia | Oligoflexales |  |  | UI |
| 71 | Actinobacteriota | Thermoleophilia | Gaiellales |  |  | UI |
| 72 | Bacteroidota | Bacteroidia | Chitinophagales | Chitinophagaceae | Dinghuibacter | UI |
| 73 | Proteobacteria | Alphaproteobacteria | Sphingomonadales | Sphingomonadaceae | Sphingomonas | ES |
| 74 | Bacteroidota | Bacteroidia | Flavobacteriales | Flavobacteriaceae | Flavobacterium | EP |
| 75 | Bacteroidota | Bacteroidia | Sphingobacteriales | env.OPS-17 | env.OPS-17 | UI |
| 76 | Gemmatimonadota | Gemmatimonadetes | Gemmatimonadales | Gemmatimonadaceae |  | TH |
| 77 | Actinobacteriota | Acidimicrobiia | Microtrichales | Ilumatobacteraceae | CL500-29-marine-group | UI |
| 78 | Acidobacteriota | Acidobacteriae | Acidobacteriae | Acidobacteriae | Paludibaculum | UI |
| 79 | Actinobacteriota | Actinobacteria | Frankiales | Sporichthyaceae |  | UI |
| 80 | Proteobacteria | Gammaproteobacteria | Burkholderiales | Comamonadaceae | Aquabacterium | BG |
| 82 | Proteobacteria | Alphaproteobacteria | Caulobacterales | Caulobacteraceae | Caulobacter | ES |
| 83 | Proteobacteria | Alphaproteobacteria | Rhodobacterales | Rhodobacteraceae | Rhodobacter | UI |
| 84 | Actinobacteriota | Actinobacteria | Micrococcales | Microbacteriaceae |  | TH |
| 85 | Proteobacteria | Gammaproteobacteria | Legionellales | Legionellaceae | Legionella | BG |
| 86 | Verrucomicrobiota | Verrucomicrobiae | Verrucomicrobiales | Rubritaleaceae | Luteolibacter | EP |
| 87 | Actinobacteriota | Actinobacteria | Frankiales | Sporichthyaceae |  | DL |
| 88 | Proteobacteria | Gammaproteobacteria | Burkholderiales | Methylophilaceae |  | EP |
| 89 | Proteobacteria | Gammaproteobacteria | Burkholderiales | Comamonadaceae |  | BG |
| 90 | Bacteroidota | Bacteroidia | Chitinophagales | Chitinophagaceae | Ferruginibacter | EP |
| 91 | Bacteroidota | Bacteroidia | Chitinophagales | Chitinophagaceae | Sediminibacterium | DL |
| 92 | Actinobacteriota | Actinobacteria | Frankiales | Sporichthyaceae |  | UI |
| 93 | Proteobacteria | Gammaproteobacteria | Burkholderiales | Comamonadaceae | Rhodoferax | EP |
| 95 | Planctomycetota | Planctomycetes | Pirellulales | Pirellulaceae |  | BG |
| 96 | Gemmatimonadota | Gemmatimonadetes | Gemmatimonadales | Gemmatimonadaceae |  | BG |
| 97 | Actinobacteriota | Actinobacteria | Frankiales | Sporichthyaceae | hgcI-clade | OE |
| 98 | Actinobacteriota | Actinobacteria | Micrococcales | Microbacteriaceae | Candidatus-Planktoluna | BG |
| 99 | Verrucomicrobiota | Verrucomicrobiae | Chthoniobacterales | Chthoniobacteraceae | Chthoniobacter | UI |
| 102 | Actinobacteriota | Actinobacteria | Micrococcales | Microbacteriaceae |  | BG |
| 103 | Proteobacteria | Alphaproteobacteria | Sphingomonadales | Sphingomonadaceae | Sphingomonas | BG |
| 104 | Proteobacteria | Alphaproteobacteria | Zavarziniales | Zavarziniaceae | Zavarziniaceae | OE |
| 105 | Verrucomicrobiota | Verrucomicrobiae | Opitutales | Opitutaceae | Opitutus | UI |
| 106 | Chloroflexi | Chloroflexia | Chloroflexales | Roseiflexaceae |  | DL |
| 107 | Bacteroidota | Bacteroidia | Chitinophagales | Chitinophagaceae | Ferruginibacter | OE |
| 108 | Bacteroidota | Bacteroidia | Sphingobacteriales | KD3-93 | KD3-93 | TH |
| 109 | Proteobacteria | Gammaproteobacteria | Burkholderiales | Comamonadaceae |  | OE |
| 110 | Actinobacteriota | Actinobacteria | Corynebacteriales | Mycobacteriaceae | Mycobacterium | BG |
| 111 | Proteobacteria | Gammaproteobacteria | Burkholderiales | Comamonadaceae |  | ES |
| 112 | Proteobacteria | Gammaproteobacteria | Burkholderiales | Comamonadaceae |  | OE |
| 113 | Verrucomicrobiota | Verrucomicrobiae | Verrucomicrobiales | Verrucomicrobiaceae | Prosthecobacter | UI |
| 114 | Verrucomicrobiota | Verrucomicrobiae | Methylacidiphilales | Methylacidiphilaceae |  | BG |
| 115 | Bacteroidota | Bacteroidia | Flavobacteriales | Crocinitomicaceae | Fluviicola | EP |
| 116 | Verrucomicrobiota | Verrucomicrobiae | Chthoniobacterales | Chthoniobacteraceae | Chthoniobacter | UI |
| 117 | Acidobacteriota | Acidobacteriae | Solibacterales | Solibacteraceae | Candidatus-Solibacter | TH |
| 119 | Planctomycetota | Planctomycetes | Isosphaerales | Isosphaeraceae |  | OE |
| 120 | Patescibacteria | Parcubacteria |  |  |  | BG |
| 122 | Proteobacteria | Alphaproteobacteria | Acetobacterales | Acetobacteraceae |  | TH |
| 123 | Bdellovibrionota | Bdellovibrionia | Bacteriovoracales | Bacteriovoracaceae | Peredibacter | BG |
| 124 | Proteobacteria | Alphaproteobacteria | Sphingomonadales | Sphingomonadaceae | Polymorphobacter | TH |
| 125 | Proteobacteria | Alphaproteobacteria | Rhizobiales | Beijerinckiaceae | FukuN57 | OE |
| 126 | Bacteroidota | Bacteroidia | Chitinophagales | Chitinophagaceae |  | EP |
| 127 | Proteobacteria | Gammaproteobacteria | Burkholderiales | TRA3-20 | TRA3-20 | UI |
| 128 | Acidobacteriota | Acidobacteriae | Solibacterales | Solibacteraceae | Candidatus-Solibacter | UI |
| 129 | Bacteroidota | Bacteroidia | Sphingobacteriales | KD3-93 | KD3-93 | BG |
| 130 | Proteobacteria | Gammaproteobacteria | Burkholderiales | Comamonadaceae | Rhodoferax | UI |
| 131 | Proteobacteria | Alphaproteobacteria | Reyranellales | Reyranellaceae | Reyranella | BG |
| 132 | Bacteroidota | Bacteroidia | Chitinophagales | Chitinophagaceae | Edaphobaculum | DL |
| 133 | Dependentiae | Babeliae | Babeliales |  |  | UI |
| 134 | Proteobacteria | Gammaproteobacteria | Piscirickettsiales | Piscirickettsiaceae | Candidatus-Endoecteinascidia | UI |
| 135 | Bacteroidota | Bacteroidia | Sphingobacteriales | env.OPS-17 | env.OPS-17 | UI |
| 136 | Proteobacteria | Gammaproteobacteria | Burkholderiales |  |  | DL |
| 137 | Proteobacteria | Gammaproteobacteria | Burkholderiales | Comamonadaceae |  | TH |
| 138 | Actinobacteriota | Actinobacteria | Frankiales | Sporichthyaceae | hgcI-clade | BG |
| 140 | Verrucomicrobiota | Verrucomicrobiae | Chthoniobacterales | Terrimicrobiaceae | FukuN18-freshwater-group | ES |
| 141 | Bacteroidota | Bacteroidia | Chitinophagales | Chitinophagaceae | Ferruginibacter | EP |
| 142 | Bacteroidota | Bacteroidia | Flavobacteriales | Flavobacteriaceae | Flavobacterium | EP |
| 143 | Proteobacteria | Gammaproteobacteria | Burkholderiales | Methylophilaceae | Methylotenera | DL |
| 145 | Proteobacteria | Gammaproteobacteria | Burkholderiales | Comamonadaceae | Limnohabitans | EP |
| 146 | Actinobacteriota | Actinobacteria |  |  |  | EP |
| 149 | Actinobacteriota | Actinobacteria |  |  |  | EP |
| 150 | Proteobacteria | Alphaproteobacteria | Reyranellales | Reyranellaceae | Reyranella | BG |
| 151 | Proteobacteria | Gammaproteobacteria | Burkholderiales | Nitrosomonadaceae | MND1 | DL |
| 152 | Bacteroidota | Bacteroidia | Sphingobacteriales | LiUU-11-161 | LiUU-11-161 | OE |
| 154 | Proteobacteria | Alphaproteobacteria |  |  |  | UI |
| 155 | Proteobacteria | Alphaproteobacteria | Acetobacterales | Acetobacteraceae |  | TH |
| 156 | Proteobacteria | Alphaproteobacteria | Rhizobiales | Beijerinckiaceae |  | EP |
| 157 | Actinobacteriota | Acidimicrobiia | Microtrichales | Microtrichaceae |  | BG |
| 158 | Actinobacteriota | Actinobacteria | PeM15 | PeM15 | PeM15 | OE |
| 159 | Bacteroidota | Bacteroidia | Cytophagales | Spirosomaceae |  | UI |
| 160 | Proteobacteria | Gammaproteobacteria | Legionellales | Legionellaceae | Legionella | DL |
| 162 | Proteobacteria | Alphaproteobacteria | Rickettsiales | Rickettsiaceae | Candidatus-Megaira | ES |
| 163 | Proteobacteria | Gammaproteobacteria | Burkholderiales | Burkholderiaceae | Polynucleobacter | EP |
| 164 | Armatimonadota | Fimbriimonadia | Fimbriimonadales | Fimbriimonadaceae | Fimbriimonadaceae | BG |
| 165 | Actinobacteriota | Acidimicrobiia |  |  |  | UI |
| 168 | Proteobacteria | Gammaproteobacteria | Burkholderiales | Nitrosomonadaceae | GOUTA6 | UI |
| 170 | Proteobacteria | Alphaproteobacteria | Sphingomonadales | Sphingomonadaceae | Parablastomonas | UI |
| 171 | Actinobacteriota | Acidimicrobiia | Microtrichales | Microtrichaceae | IMCC26207 | OE |
| 172 | Proteobacteria | Alphaproteobacteria | Paracaedibacterales | Paracaedibacteraceae | Candidatus-Captivus | UI |
| 173 | Planctomycetota | Planctomycetes | Gemmatales | Gemmataceae | Fimbriiglobus | ES |
| 174 | Proteobacteria | Alphaproteobacteria | Sphingomonadales | Sphingomonadaceae | Novosphingobium | EP |
| 175 | Proteobacteria | Alphaproteobacteria | Rhodospirillales | Rhodospirillaceae |  | UI |
| 176 | Proteobacteria | Gammaproteobacteria | Ga0077536 | Ga0077536 | Ga0077536 | BG |
| 177 | Actinobacteriota | Actinobacteria | Frankiales | Sporichthyaceae | Sporichthya | OE |
| 178 | Actinobacteriota | Acidimicrobiia |  |  |  | BG |
| 180 | Planctomycetota | Phycisphaerae | Phycisphaerales | Phycisphaeraceae | CL500-3 | UI |
| 181 | Proteobacteria | Gammaproteobacteria | Cellvibrionales | Cellvibrionaceae |  | BG |
| 182 | Actinobacteriota | Actinobacteria | PeM15 | PeM15 | PeM15 | UI |
| 183 | Proteobacteria | Alphaproteobacteria | Rhizobiales | Beijerinckiaceae |  | UI |
| 184 | Bdellovibrionota | Bdellovibrionia | Bacteriovoracales | Bacteriovoracaceae | Bacteriovorax | BG |
| 185 | Actinobacteriota | Actinobacteria | Frankiales | Sporichthyaceae |  | UI |
| 187 | Proteobacteria | Gammaproteobacteria | Burkholderiales | Comamonadaceae |  | BG |
| 188 | Proteobacteria | Gammaproteobacteria | Legionellales | Legionellaceae | Legionella | UI |
| 189 | Proteobacteria | Gammaproteobacteria | Burkholderiales | Oxalobacteraceae |  | TH |
| 190 | Bacteroidota | Bacteroidia | Chitinophagales | Chitinophagaceae | Ferruginibacter | EP |
| 191 | Actinobacteriota | Acidimicrobiia | Microtrichales | Microtrichaceae |  | UI |
| 192 | Proteobacteria | Gammaproteobacteria | Burkholderiales | Rhodocyclaceae | Georgfuchsia | UI |
| 193 | Proteobacteria | Alphaproteobacteria | Rhizobiales | Beijerinckiaceae | Methylobacterium-Methylorubrum | BG |
| 194 | Bacteroidota | Bacteroidia | Chitinophagales |  |  | BG |
| 195 | Proteobacteria | Gammaproteobacteria | Burkholderiales | Burkholderiaceae | Polynucleobacter | BG |
| 196 | Proteobacteria | Alphaproteobacteria | Caulobacterales | Caulobacteraceae | Phenylobacterium | BG |
| 197 | Proteobacteria | Alphaproteobacteria | Acetobacterales | Acetobacteraceae |  | BG |
| 200 | Planctomycetota | Planctomycetes | Pirellulales | Pirellulaceae |  | UI |
| 201 | Bacteroidota | Bacteroidia | Flavobacteriales | Crocinitomicaceae | Fluviicola | EP |
| 203 | Proteobacteria | Gammaproteobacteria | Steroidobacterales | Steroidobacteraceae |  | UI |
| 204 | Actinobacteriota | Thermoleophilia | Gaiellales |  |  | BG |
| 205 | Bacteroidota | Bacteroidia | Chitinophagales | Chitinophagaceae | Parasediminibacterium | ES |
| 207 | Actinobacteriota | Thermoleophilia | Solirubrobacterales |  |  | BG |
| 208 | Proteobacteria | Gammaproteobacteria | Burkholderiales | Nitrosomonadaceae |  | UI |
| 210 | Actinobacteriota | Acidimicrobiia | Microtrichales | Ilumatobacteraceae | CL500-29-marine-group | UI |
| 211 | Proteobacteria | Gammaproteobacteria | Burkholderiales | Methylophilaceae | Methylotenera | DL |
| 212 | Actinobacteriota | Actinobacteria | PeM15 | PeM15 | PeM15 | OE |
| 213 | Verrucomicrobiota | Verrucomicrobiae | Verrucomicrobiales | Rubritaleaceae | Luteolibacter | UI |
| 214 | Proteobacteria | Gammaproteobacteria |  |  |  | UI |
| 215 | Bacteroidota | Bacteroidia | Chitinophagales |  |  | OE |
| 216 | Proteobacteria | Gammaproteobacteria | Pseudomonadales | Pseudomonadaceae | Pseudomonas | TH |
| 218 | Proteobacteria | Gammaproteobacteria | Burkholderiales | Alcaligenaceae | Achromobacter | BG |
| 219 | Proteobacteria | Alphaproteobacteria | Paracaedibacterales | Paracaedibacteraceae |  | UI |
| 220 | Proteobacteria | Alphaproteobacteria | Micropepsales | Micropepsaceae |  | UI |
| 221 | Proteobacteria | Alphaproteobacteria | Acetobacterales | Acetobacteraceae | Rhodovastum | DL |
| 222 | Proteobacteria | Alphaproteobacteria | Acetobacterales | Acetobacteraceae | Rhodovastum | BG |
| 223 | Spirochaetota | Spirochaetia | Spirochaetales | Spirochaetaceae | Spirochaeta-2 | BG |
| 224 | Proteobacteria | Gammaproteobacteria | Burkholderiales | Comamonadaceae |  | EP |
| 225 | Proteobacteria | Gammaproteobacteria | Burkholderiales | Comamonadaceae | Rhodoferax | OE |
| 226 | Planctomycetota | Planctomycetes | Pirellulales | Pirellulaceae |  | UI |
| 228 | Bacteroidota | Bacteroidia | Flavobacteriales | Flavobacteriaceae | Flavobacterium | EP |
| 229 | Verrucomicrobiota | Verrucomicrobiae | Pedosphaerales | Pedosphaeraceae |  | UI |
| 230 | Proteobacteria | Alphaproteobacteria |  |  |  | BG |
| 231 | Bacteroidota | Bacteroidia |  |  |  | BG |
| 232 | Verrucomicrobiota | Verrucomicrobiae | Opitutales | Opitutaceae |  | BG |
| 233 | Bacteroidota | Bacteroidia | Sphingobacteriales | NS11-12-marine-group | NS11-12-marine-group | DL |
| 234 | Bacteroidota | Bacteroidia | Flavobacteriales | Crocinitomicaceae | Fluviicola | TH |
| 235 | Bacteroidota | Bacteroidia | Sphingobacteriales | KD3-93 | KD3-93 | UI |
| 236 | Myxococcota | Polyangia | Polyangiales | Polyangiaceae | Pajaroellobacter | UI |
| 237 | Verrucomicrobiota | Verrucomicrobiae | Opitutales | Opitutaceae |  | UI |
| 238 | Bacteroidota | Bacteroidia | Flavobacteriales | Crocinitomicaceae | Fluviicola | UI |
| 239 | Verrucomicrobiota | Verrucomicrobiae | Pedosphaerales | Pedosphaeraceae | SH3-11 | BG |
| 240 | Verrucomicrobiota | Verrucomicrobiae | Chthoniobacterales | Chthoniobacteraceae | Chthoniobacter | BG |
| 241 | Actinobacteriota | Acidimicrobiia |  |  |  | DL |
| 242 | Bacteroidota | Bacteroidia | Sphingobacteriales | Sphingobacteriaceae |  | BG |
| 243 | Proteobacteria | Gammaproteobacteria | Burkholderiales | Oxalobacteraceae | Janthinobacterium | BG |
| 244 | Myxococcota | Polyangia | Haliangiales | Haliangiaceae | Haliangium | UI |
| 245 | Proteobacteria | Alphaproteobacteria | SAR11-clade | Clade III | Clade III | BG |
| 247 | Proteobacteria | Alphaproteobacteria | Caulobacterales | Caulobacteraceae | Brevundimonas | DL |
| 248 | Proteobacteria | Alphaproteobacteria | Rhizobiales | Beijerinckiaceae | Methylobacterium-Methylorubrum | BG |
| 249 | Bacteroidota | Bacteroidia | Flavobacteriales | Crocinitomicaceae | Fluviicola | BG |
| 250 | Proteobacteria | Gammaproteobacteria |  |  |  | UI |
| 251 | Bacteroidota | Bacteroidia | Flavobacteriales | Flavobacteriaceae | Flavobacterium | ES |
| 252 | Proteobacteria | Gammaproteobacteria | Burkholderiales | Oxalobacteraceae |  | EP |
| 253 | Proteobacteria | Gammaproteobacteria | Burkholderiales | Chromobacteriaceae | Vogesella | EP |
| 255 | Planctomycetota | Planctomycetes | Pirellulales | Pirellulaceae | Pirellula | UI |
| 256 | Proteobacteria | Alphaproteobacteria | Caulobacterales | Caulobacteraceae | Phenylobacterium | BG |
| 257 | Myxococcota | NA |  |  |  | BG |
| 258 | Proteobacteria | Gammaproteobacteria | Burkholderiales | B1-7BS | B1-7BS | UI |
| 260 | Bdellovibrionota | Oligoflexia | Silvanigrellales | Silvanigrellaceae |  | TH |
| 261 | Actinobacteriota | Actinobacteria | Frankiales | Sporichthyaceae |  | DL |
| 262 | Actinobacteriota | Actinobacteria | Frankiales | Sporichthyaceae | hgcI-clade | EP |
| 265 | Proteobacteria | Alphaproteobacteria | Caulobacterales | Caulobacteraceae |  | BG |
| 266 | Proteobacteria | Gammaproteobacteria | Burkholderiales | Nitrosomonadaceae |  | UI |
| 267 | Actinobacteriota | Acidimicrobiia | Microtrichales | Ilumatobacteraceae | CL500-29-marine-group | UI |
| 268 | Bacteroidota | Bacteroidia | Flavobacteriales | Flavobacteriaceae | Flavobacterium | OE |
| 269 | Proteobacteria | Gammaproteobacteria | R7C24 | R7C24 | R7C24 | OE |
| 271 | Bacteroidota | Bacteroidia | Chitinophagales | Chitinophagaceae |  | DL |
| 273 | Proteobacteria | Alphaproteobacteria | Rhizobiales | Devosiaceae | Devosia | UI |
| 274 | Desulfobacterota | Desulfuromonadia | Geobacterales | Geobacteraceae | Geobacter | DL |
| 276 | Proteobacteria | Gammaproteobacteria | Burkholderiales | Oxalobacteraceae | Undibacterium | BG |
| 277 | Proteobacteria | Gammaproteobacteria | Burkholderiales | Comamonadaceae | Aquabacterium | BG |
| 278 | Proteobacteria | Gammaproteobacteria | Burkholderiales | Comamonadaceae | Polaromonas | TH |
| 279 | Proteobacteria | Alphaproteobacteria | Paracaedibacterales | Paracaedibacteraceae | Candidatus-Finniella | UI |
| 280 | Proteobacteria | Alphaproteobacteria | Rhizobiales | Beijerinckiaceae |  | UI |
| 281 | Proteobacteria | Alphaproteobacteria | Rickettsiales | Fokiniaceae |  | EP |
| 282 | Proteobacteria | Gammaproteobacteria | Burkholderiales |  |  | EP |
| 283 | Bacteroidota | Bacteroidia | Sphingobacteriales | NS11-12-marine-group | NS11-12-marine-group | DL |
| 284 | Proteobacteria | Alphaproteobacteria | Sphingomonadales | Sphingomonadaceae |  | EP |
| 285 | Bacteroidota | Bacteroidia | Cytophagales |  |  | EP |
| 288 | Proteobacteria | Gammaproteobacteria | Burkholderiales | Comamonadaceae |  | BG |
| 289 | Proteobacteria | Gammaproteobacteria | Burkholderiales | Comamonadaceae |  | DL |
| 290 | Actinobacteriota | Thermoleophilia | Solirubrobacterales | Solirubrobacteraceae |  | UI |
| 291 | Actinobacteriota | Actinobacteria | Micrococcales | Microbacteriaceae |  | EP |
| 292 | Actinobacteriota | Actinobacteria | Frankiales | Sporichthyaceae | Candidatus-Planktophila | DL |
| 293 | Myxococcota | Polyangia | mle1-27 | mle1-27 | mle1-27 | BG |
| 294 | Proteobacteria | Gammaproteobacteria | Burkholderiales | Comamonadaceae | Rhodoferax | BG |
| 295 | Dependentiae | Babeliae | Babeliales | Babeliales | Babeliales | OE |
| 297 | Proteobacteria | Gammaproteobacteria | Legionellales | Legionellaceae | Legionella | BG |
| 299 | Proteobacteria | Gammaproteobacteria | Enterobacterales | Enterobacteriaceae | Escherichia-Shigella | BG |
| 300 | Patescibacteria | Saccharimonadia | Saccharimonadales | Saccharimonadales | Saccharimonadales | BG |
| 301 | Bdellovibrionota | Oligoflexia | 0319-6G20 | 0319-6G20 | 0319-6G20 | EP |
| 302 | Planctomycetota | vadinHA49 | vadinHA49 | vadinHA49 | vadinHA49 | BG |
| 303 | Bacteroidota | Bacteroidia | Chitinophagales | Chitinophagaceae |  | OE |
| 304 | Actinobacteriota | Actinobacteria | Frankiales | Sporichthyaceae | Longivirga | BG |
| 305 | Planctomycetota | Planctomycetes | Pirellulales | Pirellulaceae |  | BG |
| 306 | Bdellovibrionota | Bdellovibrionia | Bdellovibrionales | Bdellovibrionaceae | OM27-clade | UI |
| 308 | Planctomycetota | Planctomycetes | Gemmatales | Gemmataceae | Zavarzinella | DL |
| 309 | Verrucomicrobiota | Verrucomicrobiae | Verrucomicrobiales | Rubritaleaceae | Luteolibacter | UI |
| 310 | Myxococcota | Polyangia | Polyangiales | Eel-36e1D6 | Eel-36e1D6 | UI |
| 311 | Bacteroidota | Bacteroidia | Bacteroidales | Prolixibacteraceae | BSV13 | DL |
| 313 | Bdellovibrionota | Oligoflexia | Silvanigrellales | Silvanigrellaceae | Silvanigrella | EP |
| 315 | Proteobacteria | Gammaproteobacteria | Burkholderiales | Comamonadaceae |  | TH |
| 316 | Proteobacteria | Gammaproteobacteria | Pseudomonadales | Moraxellaceae |  | BG |
| 317 | Proteobacteria | Alphaproteobacteria | Caulobacterales | Caulobacteraceae | Caulobacter | UI |
| 319 | Planctomycetota | Planctomycetes | Isosphaerales | Isosphaeraceae |  | BG |
| 320 | Actinobacteriota | Actinobacteria | PeM15 | PeM15 | PeM15 | UI |
| 321 | Proteobacteria | Gammaproteobacteria | Legionellales | Legionellaceae | Legionella | BG |
| 322 | Verrucomicrobiota | Chlamydiae | Chlamydiales | Parachlamydiaceae |  | BG |
| 323 | Proteobacteria | Alphaproteobacteria | Sphingomonadales | Sphingomonadaceae | Sphingorhabdus | OE |
| 324 | Proteobacteria | Gammaproteobacteria | Legionellales | Legionellaceae | Legionella | OE |
| 325 | Proteobacteria | Alphaproteobacteria | Acetobacterales | Acetobacteraceae | Rhodovarius | EP |
| 326 | Planctomycetota | Planctomycetes | Planctomycetales | Schlesneriaceae | Schlesneria | BG |
| 330 | Proteobacteria | Gammaproteobacteria | Pseudomonadales | Pseudomonadaceae | Pseudomonas | BG |
| 332 | Proteobacteria | Alphaproteobacteria | Sphingomonadales | Sphingomonadaceae | Sphingomonas | BG |
| 333 | Bacteroidota | Bacteroidia | Chitinophagales |  |  | DL |
| 334 | Acidobacteriota | Blastocatellia | Blastocatellales | Blastocatellaceae | Blastocatella | OE |
| 335 | NA | NA |  |  |  | DL |
| 339 | Proteobacteria | Gammaproteobacteria | Pseudomonadales | Moraxellaceae | [Agitococcus]-lubricus-group | BG |
| 340 | Bacteroidota | Bacteroidia | Chitinophagales | Chitinophagaceae |  | BG |
| 341 | Bdellovibrionota | Oligoflexia | Oligoflexales |  |  | UI |
| 342 | Proteobacteria | Gammaproteobacteria | Burkholderiales | Comamonadaceae | Rhodoferax | DL |
| 343 | Proteobacteria | Gammaproteobacteria | Burkholderiales | Comamonadaceae | Rhodoferax | TH |
| 345 | Bdellovibrionota | Oligoflexia | 053A03-B-DI-P58 | 053A03-B-DI-P58 | 053A03-B-DI-P58 | DL |
| 346 | Proteobacteria | Gammaproteobacteria | Burkholderiales | Oxalobacteraceae | Actimicrobium | TH |
| 347 | Actinobacteriota | Actinobacteria | Frankiales | Sporichthyaceae | Sporichthyaceae | OE |
| 350 | Patescibacteria | Saccharimonadia | Saccharimonadales |  |  | DL |
| 351 | Actinobacteriota | Thermoleophilia | Gaiellales |  |  | DL |
| 352 | Proteobacteria | Gammaproteobacteria | Burkholderiales | Burkholderiaceae |  | EP |
| 353 | Proteobacteria | Alphaproteobacteria | Caulobacterales | Caulobacteraceae | Phenylobacterium | UI |
| 354 | Patescibacteria | Saccharimonadia | Saccharimonadales |  |  | OE |
| 355 | Dependentiae | Babeliae | Babeliales | UBA12409 | UBA12409 | DL |
| 357 | Bacteroidota | Bacteroidia | Chitinophagales | Chitinophagaceae | Ferruginibacter | OE |
| 358 | Proteobacteria | Gammaproteobacteria | Methylococcales | Methylomonadaceae | Crenothrix | DL |
| 360 | Bdellovibrionota | Bdellovibrionia | Bdellovibrionales | Bdellovibrionaceae | Bdellovibrio | UI |
| 361 | Proteobacteria | Gammaproteobacteria | Burkholderiales | Oxalobacteraceae | Undibacterium | TH |
| 362 | Bacteroidota | Bacteroidia | Chitinophagales | 37-13 | 37-13 | DL |
| 363 | Patescibacteria | Gracilibacteria | Gracilibacteria | Gracilibacteria | Gracilibacteria | EP |
| 364 | Proteobacteria | Gammaproteobacteria | Pseudomonadales | Pseudomonadaceae | Pseudomonas | ES |
| 365 | Bacteroidota | Bacteroidia | Sphingobacteriales | NS11-12-marine-group | NS11-12-marine-group | OE |
| 367 | Actinobacteriota | Thermoleophilia | Solirubrobacterales | Solirubrobacteraceae |  | EP |
| 369 | Firmicutes | Bacilli | Lactobacillales | Enterococcaceae | Enterococcus | BG |
| 370 | Proteobacteria | Gammaproteobacteria | Burkholderiales | TRA3-20 | TRA3-20 | UI |
| 371 | Bacteroidota | Bacteroidia | Bacteroidales | Prolixibacteraceae | BSV13 | DL |
| 372 | Chloroflexi | SL56 | SL56-marine-group | SL56-marine-group | SL56-marine-group | UI |
| 373 | Proteobacteria | Gammaproteobacteria | Legionellales | Legionellaceae | Legionella | BG |
| 374 | Myxococcota | Polyangia | Polyangiales | Polyangiaceae | Aetherobacter | BG |
| 375 | Proteobacteria | Alphaproteobacteria | Rhizobiales | Beijerinckiaceae |  | BG |
| 377 | Proteobacteria | Gammaproteobacteria | Burkholderiales | Methylophilaceae | Candidatus-Methylopumilus | EP |
| 378 | Proteobacteria | Gammaproteobacteria | Enterobacterales | Enterobacteriaceae |  | EP |
| 379 | Proteobacteria | Alphaproteobacteria | Paracaedibacterales | Paracaedibacteraceae | Candidatus-Finniella | DL |
| 382 | Proteobacteria | Alphaproteobacteria | Rhodospirillales | Rhodospirillaceae |  | UI |
| 383 | Proteobacteria | Gammaproteobacteria | Burkholderiales | Nitrosomonadaceae |  | UI |
| 384 | Proteobacteria | Gammaproteobacteria | Burkholderiales | Gallionellaceae | Sideroxydans | DL |
| 385 | Proteobacteria | Alphaproteobacteria | Rickettsiales | Candidatus-Hepatincola | Candidatus-Hepatincola | DL |
| 386 | Proteobacteria | Gammaproteobacteria | Burkholderiales | Oxalobacteraceae | Undibacterium | BG |
| 387 | Proteobacteria | Gammaproteobacteria | Burkholderiales | Oxalobacteraceae |  | TH |
| 388 | Bacteroidota | Bacteroidia | Chitinophagales | Chitinophagaceae | Edaphobaculum | BG |
| 389 | Proteobacteria | Gammaproteobacteria | Burkholderiales | Comamonadaceae |  | DL |
| 390 | Proteobacteria | Gammaproteobacteria | Methylococcales | Methylomonadaceae | Crenothrix | DL |
| 391 | Desulfobacterota | Desulfuromonadia | Geobacterales | Geobacteraceae | Geobacter | DL |
| 392 | Bacteroidota | Bacteroidia | Chitinophagales | Chitinophagaceae | Edaphobaculum | BG |
| 393 | Firmicutes | Clostridia | Peptostreptococcales-Tissierellales | Peptostreptococcales-Tissierellales | Finegoldia | BG |
| 396 | Actinobacteriota | Actinobacteria | Micrococcales | Microbacteriaceae |  | EP |
| 397 | Bacteroidota | Bacteroidia | Sphingobacteriales | NS11-12-marine-group | NS11-12-marine-group | EP |
| 398 | Bacteroidota | Bacteroidia | Cytophagales | Microscillaceae |  | ES |
| 399 | Proteobacteria | Gammaproteobacteria | Burkholderiales | Comamonadaceae | Curvibacter | BG |
| 401 | Proteobacteria | Gammaproteobacteria | Burkholderiales |  |  | UI |
| 402 | Campilobacterota | Campylobacteria | Campylobacterales | Sulfurimonadaceae | Sulfuricurvum | DL |
| 403 | Armatimonadota | Armatimonadia | Armatimonadales | Armatimonadales | Armatimonadales | BG |
| 404 | Bacteroidota | Bacteroidia | Chitinophagales | Chitinophagaceae |  | OE |
| 405 | Bdellovibrionota | Oligoflexia | Oligoflexales |  |  | DL |
| 406 | Patescibacteria | Saccharimonadia | Saccharimonadales | Saccharimonadales | Saccharimonadales | OE |
| 407 | Patescibacteria | Saccharimonadia | Saccharimonadales | Saccharimonadales | Saccharimonadales | DL |
| 408 | Proteobacteria | Gammaproteobacteria | Burkholderiales | Comamonadaceae | Polaromonas | TH |
| 409 | Proteobacteria | Gammaproteobacteria | Burkholderiales | Oxalobacteraceae |  | TH |
| 410 | Proteobacteria | Alphaproteobacteria | Sphingomonadales | Sphingomonadaceae | Sphingomonas | BG |
| 411 | Actinobacteriota | Acidimicrobiia |  |  |  | OE |
| 412 | Bacteroidota | Bacteroidia | Chitinophagales | Chitinophagaceae | Dinghuibacter | EP |
| 414 | Bacteroidota | Bacteroidia | Chitinophagales | Chitinophagaceae | Ferruginibacter | BG |
| 415 | Bdellovibrionota | Oligoflexia | Oligoflexales |  |  | BG |
| 417 | Proteobacteria | Alphaproteobacteria | Sphingomonadales | Sphingomonadaceae | Novosphingobium | ES |
| 419 | Proteobacteria | Gammaproteobacteria | Pseudomonadales | Pseudomonadaceae | Pseudomonas | BG |
| 420 | Actinobacteriota | Actinobacteria | Frankiales | Sporichthyaceae | hgcI-clade | BG |
| 424 | Proteobacteria | Alphaproteobacteria | Rickettsiales | Rickettsiaceae |  | DL |
| 425 | Proteobacteria | Gammaproteobacteria | Burkholderiales | Comamonadaceae | Rhizobacter | UI |
| 426 | Actinobacteriota | Actinobacteria | Frankiales | Sporichthyaceae | Longivirga | BG |
| 427 | Patescibacteria | Saccharimonadia | Saccharimonadales | Saccharimonadales | Saccharimonadales | DL |
| 428 | Proteobacteria | Alphaproteobacteria | Acetobacterales | Acetobacteraceae | Rhodovastum | BG |
| 429 | Bacteroidota | Bacteroidia | Chitinophagales | Chitinophagaceae |  | OE |
| 430 | Actinobacteriota | Actinobacteria | Micrococcales | Micrococcaceae | Micrococcus | BG |
| 431 | Proteobacteria | Alphaproteobacteria | Rhizobiales | Xanthobacteraceae |  | BG |
| 433 | Proteobacteria | Alphaproteobacteria | Rickettsiales | Rickettsiaceae |  | DL |
| 437 | Proteobacteria | Alphaproteobacteria | Sphingomonadales | Sphingomonadaceae | Novosphingobium | EP |
| 438 | Proteobacteria | Gammaproteobacteria | Burkholderiales | Comamonadaceae | Rhodoferax | DL |
| 439 | Proteobacteria | Alphaproteobacteria | Sphingomonadales | Sphingomonadaceae | Sphingobium | BG |
| 441 | Proteobacteria | Gammaproteobacteria | Salinisphaerales | Solimonadaceae |  | BG |
| 442 | Proteobacteria | Gammaproteobacteria | Salinisphaerales | Solimonadaceae | Nevskia | BG |
| 446 | Bacteroidota | Bacteroidia | Flavobacteriales | Flavobacteriaceae | Flavobacterium | TH |
| 447 | Bdellovibrionota | Oligoflexia | 0319-6G20 | 0319-6G20 | 0319-6G20 | BG |
| 448 | Bdellovibrionota | Oligoflexia | Oligoflexales |  |  | BG |
| 449 | Bacteroidota | Bacteroidia | Bacteroidales | Prolixibacteraceae | BSV13 | DL |
| 450 | Proteobacteria | Gammaproteobacteria | Burkholderiales | Oxalobacteraceae | Actimicrobium | TH |
| 451 | Bacteroidota | Bacteroidia | Bacteroidales | Prolixibacteraceae | BSV13 | DL |
| 452 | Proteobacteria | Gammaproteobacteria | Burkholderiales | Gallionellaceae |  | DL |
| 453 | Proteobacteria | Gammaproteobacteria | Pseudomonadales | Moraxellaceae | Enhydrobacter | OE |
| 454 | Bacteroidota | Bacteroidia | Chitinophagales | Chitinophagaceae |  | BG |
| 455 | Proteobacteria | Alphaproteobacteria | Acetobacterales | Acetobacteraceae |  | BG |
| 457 | Proteobacteria | Gammaproteobacteria | Legionellales | Legionellaceae | Legionella | DL |
| 458 | Verrucomicrobiota | Verrucomicrobiae |  |  |  | TH |
| 459 | Actinobacteriota | Actinobacteria | PeM15 | PeM15 | PeM15 | BG |
| 461 | Actinobacteriota | Actinobacteria |  |  |  | EP |
| 464 | Firmicutes | Bacilli | Lactobacillales | Streptococcaceae | Streptococcus | OE |
| 466 | Bacteroidota | Bacteroidia | Cytophagales | Spirosomaceae | Arcicella | TH |
| 467 | Bacteroidota | Bacteroidia | Sphingobacteriales | env.OPS-17 | env.OPS-17 | BG |
| 469 | Proteobacteria | Gammaproteobacteria | Legionellales | Legionellaceae | Legionella | BG |
| 470 | Actinobacteriota | Actinobacteria | Frankiales | Sporichthyaceae |  | DL |
| 471 | Verrucomicrobiota | Verrucomicrobiae | Chthoniobacterales | Chthoniobacteraceae | Chthoniobacter | BG |
| 473 | Proteobacteria | Alphaproteobacteria | Rickettsiales | Fokiniaceae |  | OE |
| 474 | Bacteroidota | Bacteroidia | Flavobacteriales | Flavobacteriaceae | Flavobacterium | BG |
| 475 | Proteobacteria | Gammaproteobacteria | Burkholderiales | Oxalobacteraceae | Undibacterium | TH |
| 476 | Proteobacteria | Gammaproteobacteria | Burkholderiales | Comamonadaceae | Rhodoferax | DL |
| 477 | Proteobacteria | Alphaproteobacteria | Rickettsiales |  |  | ES |
| 479 | Actinobacteriota | Actinobacteria | Micrococcales | Micrococcaceae |  | DL |
| 480 | Proteobacteria | Gammaproteobacteria |  |  |  | DL |
| 481 | Campilobacterota | Campylobacteria | Campylobacterales | Sulfurimonadaceae | Sulfurimonas | DL |
| 483 | Proteobacteria | Gammaproteobacteria | Burkholderiales | Oxalobacteraceae |  | TH |
| 484 | Patescibacteria | Saccharimonadia | Saccharimonadales | Saccharimonadales | Saccharimonadales | DL |
| 485 | Proteobacteria | Gammaproteobacteria | Burkholderiales | Oxalobacteraceae |  | TH |
| 486 | Proteobacteria | Alphaproteobacteria | Caulobacterales | Caulobacteraceae | Brevundimonas | DL |
| 487 | Actinobacteriota | Thermoleophilia | Solirubrobacterales | Solirubrobacteraceae | Patulibacter | BG |
| 488 | Proteobacteria | Gammaproteobacteria | Burkholderiales | Rhodocyclaceae |  | DL |
| 489 | Proteobacteria | Gammaproteobacteria | Methylococcales | Methylomonadaceae |  | DL |
| 490 | Patescibacteria | Saccharimonadia | Saccharimonadales | LWQ8 | LWQ8 | BG |
| 491 | Proteobacteria | Alphaproteobacteria | Rhizobiales | Xanthobacteraceae | Rhodopseudomonas | BG |
| 492 | Bdellovibrionota | Oligoflexia | Oligoflexales |  |  | BG |
| 493 | Proteobacteria | Alphaproteobacteria | Rickettsiales | Rickettsiaceae |  | DL |
| 495 | Proteobacteria | Gammaproteobacteria | Gammaproteobacteria-Incertae-Sedis | Unknown-Family | Unknown-Family | BG |
| 497 | Bacteroidota | Bacteroidia | Chitinophagales | Chitinophagaceae | Edaphobaculum | BG |
| 498 | Proteobacteria | Alphaproteobacteria | Rhizobiales | Hyphomicrobiaceae | Hyphomicrobium | BG |
| 500 | Bacteroidota | Bacteroidia | Chitinophagales | Chitinophagaceae | Ferruginibacter | DL |
| 501 | Bacteroidota | Bacteroidia | Chitinophagales | Chitinophagaceae | Edaphobaculum | DL |
| 504 | Proteobacteria | Alphaproteobacteria | Rickettsiales | Rickettsiaceae |  | DL |
| 505 | Armatimonadota | Chthonomonadetes | Chthonomonadales | Chthonomonadaceae | Chthonomonas | DL |
| 508 | Proteobacteria | Gammaproteobacteria | Burkholderiales | Comamonadaceae |  | EP |
| 509 | Proteobacteria | Alphaproteobacteria | Acetobacterales | Acetobacteraceae | Roseomonas | EP |
| 510 | Proteobacteria | Gammaproteobacteria | Methylococcales | Methylomonadaceae |  | BG |
| 511 | Bacteroidota | Bacteroidia |  |  |  | TH |
| 512 | Actinobacteriota | Actinobacteria | Corynebacteriales | Nocardiaceae | Rhodococcus | BG |
| 513 | Fibrobacterota | Fibrobacteria | Fibrobacterales | Fibrobacteraceae |  | BG |
| 514 | Bacteroidota | Bacteroidia | Flavobacteriales | Crocinitomicaceae | Fluviicola | BG |
| 519 | Proteobacteria | Gammaproteobacteria | Pseudomonadales | Moraxellaceae | Alkanindiges | BG |
| 520 | Proteobacteria | Gammaproteobacteria | Burkholderiales | Comamonadaceae | Variovorax | BG |
| 523 | Bacteroidota | Bacteroidia | Chitinophagales | Chitinophagaceae |  | BG |
| 525 | Proteobacteria | Alphaproteobacteria | Rhizobiales | Xanthobacteraceae | Pseudolabrys | BG |
| 526 | Patescibacteria | Parcubacteria | Candidatus-Jorgensenbacteria | Candidatus-Jorgensenbacteria | Candidatus-Jorgensenbacteria | DL |
| 528 | Firmicutes | Bacilli | Mycoplasmatales | Mycoplasmataceae |  | DL |
| 533 | Proteobacteria | Gammaproteobacteria | Burkholderiales | T34 | T34 | UI |
| 534 | Proteobacteria | Gammaproteobacteria | Cellvibrionales | Cellvibrionaceae | Cellvibrio | BG |
| 535 | Proteobacteria | Alphaproteobacteria | Caulobacterales | Caulobacteraceae | Phenylobacterium | BG |
| 537 | Proteobacteria | Gammaproteobacteria | Burkholderiales | Comamonadaceae | Aquabacterium | BG |
| 539 | Firmicutes | Clostridia | Peptostreptococcales-Tissierellales | Peptostreptococcales-Tissierellales | Fenollaria | BG |
| 540 | Proteobacteria | Alphaproteobacteria | Rickettsiales | SM2D12 | SM2D12 | BG |
| 541 | Bacteroidota | Bacteroidia | Chitinophagales | Chitinophagaceae | Edaphobaculum | BG |
| 546 | Bdellovibrionota | Oligoflexia | 0319-6G20 | 0319-6G20 | 0319-6G20 | BG |
| 550 | Actinobacteriota | Thermoleophilia | Solirubrobacterales | Solirubrobacteraceae | Conexibacter | BG |
| 551 | Firmicutes | Bacilli | Bacillales | Bacillaceae | Bacillus | BG |
| 552 | Acidobacteriota | Holophagae | Holophagales | Holophagaceae | Geothrix | DL |
| 553 | Bacteroidota | Bacteroidia | Sphingobacteriales | Sphingobacteriaceae |  | BG |
| 555 | Planctomycetota | Planctomycetes | Pirellulales | Pirellulaceae |  | BG |
| 556 | Proteobacteria | Gammaproteobacteria | CHAB-XI-27 | CHAB-XI-27 | CHAB-XI-27 | OE |
| 557 | Actinobacteriota | Actinobacteria | Frankiales | Sporichthyaceae |  | DL |
| 558 | Bacteroidota | Bacteroidia | Chitinophagales | Chitinophagaceae | Sediminibacterium | DL |
| 559 | Proteobacteria | Alphaproteobacteria | Sphingomonadales | Sphingomonadaceae | Sphingomonas | OE |
| 560 | Proteobacteria | Alphaproteobacteria | Rhizobiales | Xanthobacteraceae | Afipia | BG |
| 562 | Verrucomicrobiota | Verrucomicrobiae | Opitutales | Opitutaceae | Lacunisphaera | DL |
| 563 | Myxococcota | Myxococcia | Myxococcales | Anaeromyxobacteraceae | Anaeromyxobacter | DL |
| 564 | Proteobacteria | Gammaproteobacteria | Burkholderiales | Comamonadaceae | Rhodoferax | DL |
| 565 | Bacteroidota | Bacteroidia | Chitinophagales | Chitinophagaceae |  | DL |
| 566 | Bdellovibrionota | Oligoflexia | 0319-6G20 | 0319-6G20 | 0319-6G20 | TH |
| 567 | Campilobacterota | Campylobacteria | Campylobacterales |  |  | DL |
| 569 | Patescibacteria | Saccharimonadia | Saccharimonadales |  |  | DL |
| 570 | Bacteroidota | Bacteroidia | Cytophagales | Hymenobacteraceae | Hymenobacter | ES |
| 571 | Proteobacteria | Gammaproteobacteria | Burkholderiales | Oxalobacteraceae | Actimicrobium | TH |
| 572 | Proteobacteria | Alphaproteobacteria | Rhizobiales | Beijerinckiaceae | Methylobacterium-Methylorubrum | BG |
| 573 | Proteobacteria | Alphaproteobacteria | Rickettsiales | Rickettsiaceae |  | BG |
| 574 | Proteobacteria | Gammaproteobacteria | Xanthomonadales | Xanthomonadaceae | Stenotrophomonas | BG |
| 578 | Acidobacteriota | Holophagae | Holophagales | Holophagaceae | Geothrix | DL |
| 579 | Proteobacteria | Gammaproteobacteria | Burkholderiales | Comamonadaceae | Rhodoferax | DL |
| 580 | Patescibacteria | CPR2 | CPR2 | CPR2 | CPR2 | DL |
| 581 | Bdellovibrionota | Oligoflexia | 0319-6G20 | 0319-6G20 | 0319-6G20 | BG |
| 582 | Proteobacteria | Alphaproteobacteria | Acetobacterales | Acetobacteraceae |  | ES |
| 584 | NA | NA |  |  |  | EP |
| 585 | Firmicutes | Bacilli | Lactobacillales | Streptococcaceae | Streptococcus | BG |
| 586 | Firmicutes | Bacilli | Staphylococcales | Staphylococcaceae | Staphylococcus | BG |
| 587 | Proteobacteria | Gammaproteobacteria | Burkholderiales | Oxalobacteraceae | Herminiimonas | BG |
| 588 | Nitrospirota | Nitrospiria | Nitrospirales | Nitrospiraceae | Nitrospira | DL |
| 589 | Actinobacteriota | Actinobacteria | Corynebacteriales | Corynebacteriaceae | Corynebacterium | BG |
| 590 | Proteobacteria | Alphaproteobacteria | Rickettsiales | Rickettsiaceae |  | BG |
| 591 | Proteobacteria | Alphaproteobacteria | Caulobacterales | Caulobacteraceae | Brevundimonas | BG |
| 593 | Actinobacteriota | Actinobacteria | Micrococcales | Microbacteriaceae |  | BG |
| 596 | Bacteroidota | Bacteroidia | Chitinophagales | Saprospiraceae | Haliscomenobacter | EP |
| 597 | Proteobacteria | Gammaproteobacteria | Burkholderiales | Comamonadaceae | Rhodoferax | EP |
| 598 | Actinobacteriota | Actinobacteria | Micrococcales | Microbacteriaceae | Candidatus-Planktoluna | BG |
| 599 | Bacteroidota | Bacteroidia | Chitinophagales | Chitinophagaceae | Sediminibacterium | BG |
| 600 | Proteobacteria | Alphaproteobacteria | Sphingomonadales | Sphingomonadaceae | Sphingomonas | BG |
| 601 | Patescibacteria | Parcubacteria | Candidatus-Jorgensenbacteria | Candidatus-Jorgensenbacteria | Candidatus-Jorgensenbacteria | DL |
| 603 | Proteobacteria | Gammaproteobacteria | Burkholderiales | Oxalobacteraceae | Undibacterium | BG |
| 604 | Proteobacteria | Alphaproteobacteria | Rhizobiales | Devosiaceae | Devosia | BG |
| 607 | Proteobacteria | Gammaproteobacteria | Burkholderiales | Comamonadaceae |  | ES |
| 608 | Proteobacteria | Alphaproteobacteria | Sphingomonadales | Sphingomonadaceae | Sphingomonas | BG |
| 609 | Proteobacteria | Gammaproteobacteria | Burkholderiales | Oxalobacteraceae | Undibacterium | BG |
| 611 | Bdellovibrionota | Bdellovibrionia | Bdellovibrionales | Bdellovibrionaceae | Bdellovibrio | BG |
| 612 | Proteobacteria | Gammaproteobacteria | Pseudomonadales | Moraxellaceae | Acinetobacter | BG |
| 614 | Bacteroidota | Bacteroidia | Cytophagales | Cytophagaceae | Cytophaga | BG |
| 615 | Bacteroidota | Bacteroidia | Sphingobacteriales | env.OPS-17 | env.OPS-17 | BG |
| 616 | Proteobacteria | Alphaproteobacteria | Rickettsiales | Rickettsiaceae |  | BG |
| 617 | Bdellovibrionota | Bdellovibrionia | Bacteriovoracales | Bacteriovoracaceae | Bacteriovorax | DL |
| 618 | Verrucomicrobiota | Verrucomicrobiae | Verrucomicrobiales | Rubritaleaceae | Luteolibacter | UI |
| 623 | Proteobacteria | Gammaproteobacteria |  |  |  | DL |
| 625 | Actinobacteriota | Acidimicrobiia | IMCC26256 | IMCC26256 | IMCC26256 | BG |
| 629 | Proteobacteria | Gammaproteobacteria | Burkholderiales | Chitinibacteraceae | Iodobacter | DL |
| 635 | Bacteroidota | Bacteroidia | Sphingobacteriales | env.OPS-17 | env.OPS-17 | DL |
| 639 | Bdellovibrionota | Oligoflexia | Silvanigrellales | Silvanigrellaceae | Silvanigrella | BG |
| 643 | Proteobacteria | Gammaproteobacteria | Burkholderiales | Oxalobacteraceae |  | TH |
| 644 | Bacteroidota | Bacteroidia | Chitinophagales | Chitinophagaceae | Edaphobaculum | BG |
| 645 | Proteobacteria | Alphaproteobacteria | Sphingomonadales | Sphingomonadaceae | Rhizorhapis | TH |
| 646 | Proteobacteria | Gammaproteobacteria | Pseudomonadales | Pseudomonadaceae | Pseudomonas | BG |
| 650 | Proteobacteria | Gammaproteobacteria | Burkholderiales | Oxalobacteraceae | Undibacterium | DL |
| 651 | Bacteroidota | Bacteroidia | Flavobacteriales | Flavobacteriaceae | Flavobacterium | DL |
| 653 | Actinobacteriota | Actinobacteria | Micrococcales | Microbacteriaceae |  | OE |
| 654 | Verrucomicrobiota | Chlamydiae | Chlamydiales | Parachlamydiaceae | Neochlamydia | BG |
| 656 | Bacteroidota | Bacteroidia | Flavobacteriales | Flavobacteriaceae | Flavobacterium | BG |
| 666 | Proteobacteria | Gammaproteobacteria | Gammaproteobacteria-Incertae-Sedis | Unknown-Family | Candidatus-Ovatusbacter | BG |
| 669 | Myxococcota | Polyangia | Polyangiales |  |  | BG |
| 677 | Proteobacteria | Alphaproteobacteria | Rickettsiales | Rickettsiaceae |  | DL |
| 678 | Bacteroidota | Bacteroidia | Sphingobacteriales |  |  | DL |
| 680 | Verrucomicrobiota | Verrucomicrobiae | Verrucomicrobiales | Rubritaleaceae | Luteolibacter | DL |
| 681 | Verrucomicrobiota | Verrucomicrobiae | Verrucomicrobiales | Rubritaleaceae | Luteolibacter | DL |
| 682 | Dependentiae | Babeliae | Babeliales |  |  | DL |
| 684 | Bacteroidota | Bacteroidia | Bacteroidales | Prolixibacteraceae | BSV13 | DL |
| 685 | Proteobacteria | Gammaproteobacteria | Burkholderiales |  |  | DL |
| 686 | Desulfobacterota | Desulfobacteria | Desulfobacterales | Desulfosarcinaceae | Desulfatirhabdium | DL |
| 687 | Patescibacteria | NA |  |  |  | DL |
| 688 | Bacteroidota | Bacteroidia | Bacteroidales | Prolixibacteraceae | WCHB1-32 | DL |
| 689 | Bacteroidota | Bacteroidia | Bacteroidales | Rikenellaceae |  | DL |
| 690 | Bacteroidota | Bacteroidia | Sphingobacteriales | Lentimicrobiaceae | Lentimicrobiaceae | DL |
| 692 | Proteobacteria | Alphaproteobacteria | Rhizobiales | Xanthobacteraceae | Tardiphaga | BG |
| 693 | Bacteroidota | Bacteroidia | Chitinophagales | 37-13 | 37-13 | DL |
| 696 | Bacteroidota | Bacteroidia | Flavobacteriales | Flavobacteriaceae | Flavobacterium | TH |
| 698 | Bacteroidota | Bacteroidia | Sphingobacteriales | env.OPS-17 | env.OPS-17 | BG |
| 700 | Bdellovibrionota | Oligoflexia | Silvanigrellales | Silvanigrellaceae | Silvanigrella | BG |
| 702 | Proteobacteria | Gammaproteobacteria | Coxiellales | Coxiellaceae | Coxiella | DL |
| 703 | Proteobacteria | Alphaproteobacteria | Rickettsiales | Rickettsiaceae |  | BG |
| 706 | Patescibacteria | Saccharimonadia | Saccharimonadales | Saccharimonadales | Saccharimonadales | BG |
| 708 | Proteobacteria | Gammaproteobacteria | Burkholderiales | Oxalobacteraceae |  | DL |
| 713 | Proteobacteria | Gammaproteobacteria | Burkholderiales | Rhodocyclaceae | Sulfuritalea | DL |
| 714 | Proteobacteria | Gammaproteobacteria | Burkholderiales | Methylophilaceae |  | DL |
| 716 | Proteobacteria | Gammaproteobacteria | Burkholderiales | Oxalobacteraceae | [Aquaspirillum]-arcticum-group | TH |
| 719 | Verrucomicrobiota | Verrucomicrobiae | Verrucomicrobiales | Verrucomicrobiaceae |  | BG |
| 723 | Bacteroidota | Bacteroidia | Flavobacteriales | Crocinitomicaceae | Fluviicola | BG |
| 724 | Bacteroidota | Bacteroidia | Flavobacteriales | Crocinitomicaceae | Fluviicola | EP |
| 725 | Proteobacteria | Alphaproteobacteria | Holosporales | Holosporaceae |  | BG |
| 729 | Proteobacteria | Alphaproteobacteria | Caulobacterales | Caulobacteraceae | Brevundimonas | BG |
| 735 | Verrucomicrobiota | Verrucomicrobiae | Chthoniobacterales | Terrimicrobiaceae | Terrimicrobium | DL |
| 736 | Verrucomicrobiota | Verrucomicrobiae | Pedosphaerales | Pedosphaeraceae |  | DL |
| 741 | Proteobacteria | Alphaproteobacteria | Rhizobiales | Xanthobacteraceae |  | BG |
| 742 | Proteobacteria | Alphaproteobacteria | Holosporales | Holosporaceae |  | DL |
| 743 | Bacteroidota | Bacteroidia | Sphingobacteriales | env.OPS-17 | env.OPS-17 | DL |
| 744 | Planctomycetota | Planctomycetes | Planctomycetales | Schlesneriaceae | Schlesneria | DL |
| 745 | Fusobacteriota | Fusobacteriia | Fusobacteriales | Fusobacteriaceae | Cetobacterium | DL |
| 747 | Proteobacteria | Gammaproteobacteria | Methylococcales | Methylomonadaceae | Methylobacter | BG |
| 748 | Acidobacteriota | Holophagae | Holophagales | Holophagaceae | Geothrix | DL |
| 749 | Proteobacteria | Gammaproteobacteria | Burkholderiales | Comamonadaceae |  | TH |
| 767 | Patescibacteria | Parcubacteria |  |  |  | DL |
| 769 | Proteobacteria | Gammaproteobacteria | Methylococcales | Methylomonadaceae | Crenothrix | DL |
| 770 | Actinobacteriota | Actinobacteria | Micrococcales | Micrococcaceae | Kocuria | BG |
| 771 | Desulfobacterota | Desulfuromonadia | Geobacterales | Geobacteraceae | Geobacter | DL |
| 778 | Proteobacteria | Alphaproteobacteria | Rhizobiales | Beijerinckiaceae | Methylocella | ES |
| 781 | Proteobacteria | Gammaproteobacteria | Pseudomonadales | Pseudomonadaceae | Pseudomonas | BG |
| 782 | Dependentiae | Babeliae | Babeliales |  |  | BG |
| 785 | Proteobacteria | Alphaproteobacteria | Rickettsiales |  |  | EP |
| 798 | Firmicutes | Bacilli | Lactobacillales | Carnobacteriaceae | Granulicatella | BG |
| 801 | Bacteroidota | Bacteroidia | Cytophagales | Spirosomaceae |  | BG |
| 806 | Proteobacteria | Alphaproteobacteria | Rickettsiales | Rickettsiaceae |  | BG |
| 807 | Proteobacteria | Alphaproteobacteria | Rhodobacterales | Rhodobacteraceae | Paracoccus | BG |
| 808 | Bdellovibrionota | Bdellovibrionia | Bacteriovoracales | Bacteriovoracaceae | Peredibacter | BG |
| 809 | Actinobacteriota | Actinobacteria | Bifidobacteriales | Bifidobacteriaceae | Bifidobacterium | BG |
| 810 | Firmicutes | Bacilli | Bacillales | Planococcaceae |  | BG |
| 811 | Firmicutes | Bacilli | Bacillales | Bacillaceae | Bacillus | BG |
| 812 | Bacteroidota | Bacteroidia | Flavobacteriales | Flavobacteriaceae | Flavobacterium | BG |
| 814 | Firmicutes | Clostridia | Peptostreptococcales-Tissierellales | Peptostreptococcales-Tissierellales | Anaerococcus | BG |
| 819 | Proteobacteria | Gammaproteobacteria | Burkholderiales | Neisseriaceae |  | BG |
| 820 | Actinobacteriota | Actinobacteria | Corynebacteriales | Corynebacteriaceae | Corynebacterium | BG |
| 822 | Proteobacteria | Alphaproteobacteria | Paracaedibacterales | Paracaedibacteraceae |  | BG |
| 827 | Proteobacteria | Gammaproteobacteria | Pseudomonadales | Moraxellaceae | Alkanindiges | BG |
| 829 | Actinobacteriota | Actinobacteria | Corynebacteriales | Nocardiaceae | Rhodococcus | BG |
| 830 | Firmicutes | Clostridia | Peptostreptococcales-Tissierellales | Peptostreptococcales-Tissierellales | Peptoniphilus | BG |
| 831 | Actinobacteriota | Actinobacteria | Micrococcales | Micrococcaceae | Rothia | BG |
| 835 | Verrucomicrobiota | Verrucomicrobiae | Chthoniobacterales | Chthoniobacteraceae | Chthoniobacter | BG |
| 852 | Proteobacteria | Alphaproteobacteria | Reyranellales | Reyranellaceae | Reyranella | BG |
| 858 | Fusobacteriota | Fusobacteriia | Fusobacteriales | Fusobacteriaceae | Cetobacterium | DL |
| 862 | Patescibacteria | Parcubacteria | Candidatus-Yanofskybacteria | Candidatus-Yanofskybacteria | Candidatus-Yanofskybacteria | DL |
| 869 | Proteobacteria | Gammaproteobacteria | Burkholderiales | Comamonadaceae |  | OE |
| 880 | Patescibacteria | Parcubacteria | Parcubacteria | Parcubacteria | Parcubacteria | DL |
| 890 | Proteobacteria | Alphaproteobacteria | Reyranellales | Reyranellaceae | Reyranella | BG |
| 899 | Verrucomicrobiota | Verrucomicrobiae |  |  |  | UI |
| 905 | Bacteroidota | Bacteroidia | Chitinophagales | Chitinophagaceae | Sediminibacterium | DL |
| 920 | Proteobacteria | Alphaproteobacteria | Rickettsiales | Rickettsiaceae | Rickettsia | DL |
| 940 | Proteobacteria | Gammaproteobacteria | Burkholderiales | Oxalobacteraceae | Undibacterium | BG |
| 950 | Bacteroidota | Bacteroidia | Chitinophagales | Chitinophagaceae | Sediminibacterium | UI |
| 987 | Actinobacteriota | Actinobacteria | Frankiales | Sporichthyaceae |  | BG |
| 1005 | Actinobacteriota | Actinobacteria | Frankiales | Sporichthyaceae | hgcI-clade | BG |
| 1015 | Elusimicrobiota | Endomicrobia | Endomicrobiales | Endomicrobiaceae | Endomicrobium | DL |
| 1019 | Proteobacteria | Alphaproteobacteria | Sphingomonadales | Sphingomonadaceae |  | BG |
| 1024 | Proteobacteria | Gammaproteobacteria | Legionellales | Legionellaceae | Legionella | BG |
| 1041 | Patescibacteria | Parcubacteria | Candidatus-Yanofskybacteria | Candidatus-Yanofskybacteria | Candidatus-Yanofskybacteria | DL |
| 1048 | Patescibacteria | Parcubacteria | Candidatus-Yanofskybacteria | Candidatus-Yanofskybacteria | Candidatus-Yanofskybacteria | DL |
| 1056 | Actinobacteriota | Acidimicrobiia | Microtrichales |  |  | DL |
| 1075 | Patescibacteria | Saccharimonadia | Saccharimonadales | Saccharimonadales | Saccharimonadales | DL |
| 1085 | Actinobacteriota | Actinobacteria | Frankiales | Sporichthyaceae |  | BG |
| 1088 | Patescibacteria | Saccharimonadia | Saccharimonadales | Saccharimonadaceae |  | TH |
| 1108 | Proteobacteria | Gammaproteobacteria | Legionellales | Legionellaceae | Legionella | BG |
| 1109 | Actinobacteriota | Actinobacteria | PeM15 | PeM15 | PeM15 | BG |
| 1134 | Proteobacteria | Alphaproteobacteria | Rhizobiales | Hyphomicrobiaceae |  | BG |
| 1137 | Myxococcota | Myxococcia | Myxococcales | Myxococcaceae | P3OB-42 | DL |
| 1204 | Proteobacteria | Alphaproteobacteria | Acetobacterales | Acetobacteraceae | Roseomonas | OE |
| 1219 | Proteobacteria | Gammaproteobacteria | Pseudomonadales | Moraxellaceae | Psychrobacter | BG |
| 1222 | Desulfobacterota | Desulfuromonadia | Geobacterales | Geobacteraceae | Geobacter | DL |
| 1227 | Bacteroidota | Bacteroidia | Sphingobacteriales | Lentimicrobiaceae | Lentimicrobiaceae | DL |
| 1229 | Bacteroidota | Bacteroidia | Bacteroidales | Prolixibacteraceae | BSV13 | DL |
| 1247 | Bacteroidota | Bacteroidia | Bacteroidales | Prolixibacteraceae | BSV13 | DL |
| 1249 | Patescibacteria | Parcubacteria | Parcubacteria | Parcubacteria | Parcubacteria | DL |
| 1286 | Proteobacteria | Alphaproteobacteria | Rhodobacterales | Rhodobacteraceae |  | BG |
| 1291 | Proteobacteria | Gammaproteobacteria | Burkholderiales | Oxalobacteraceae |  | TH |
| 1304 | Firmicutes | Clostridia | Clostridiales | Clostridiaceae |  | BG |
| 1305 | Bacteroidota | Bacteroidia | Chitinophagales | Chitinophagaceae | Ferruginibacter | BG |
| 1307 | Bacteroidota | Bacteroidia | Cytophagales | Hymenobacteraceae | Hymenobacter | BG |
| 1308 | Firmicutes | Bacilli | Bacillales | Bacillaceae | Bacillus | BG |
| 1355 | Bacteroidota | Bacteroidia | Flavobacteriales | Flavobacteriaceae | Flavobacterium | BG |
| 1415 | Acidobacteriota | Blastocatellia | Blastocatellales | Blastocatellaceae | Blastocatella | BG |
| 1428 | Bacteroidota | Bacteroidia | Cytophagales | Spirosomaceae | Spirosoma | OE |
| 1452 | Proteobacteria | Alphaproteobacteria | Sphingomonadales | Sphingomonadaceae |  | BG |
| 1455 | Dependentiae | Babeliae | Babeliales |  |  | DL |
| 1461 | Proteobacteria | Alphaproteobacteria | Paracaedibacterales | Paracaedibacteraceae | Candidatus-Paracaedibacter | BG |
| 1496 | Proteobacteria | Alphaproteobacteria | Rhizobiales | Xanthobacteraceae | Rhodopseudomonas | BG |
| 1497 | Actinobacteriota | Actinobacteria | Micrococcales | Micrococcaceae | Kocuria | BG |
| 1498 | Bacteroidota | Bacteroidia | Sphingobacteriales | Sphingobacteriaceae | Solitalea | ES |
| 1506 | Bacteroidota | Bacteroidia | Chitinophagales | Chitinophagaceae | Ferruginibacter | TH |
| 1521 | Proteobacteria | Alphaproteobacteria | Rickettsiales | SM2D12 | SM2D12 | BG |
| 1531 | Proteobacteria | Gammaproteobacteria | Methylococcales | Methylomonadaceae | Crenothrix | DL |
| 1534 | NA | NA |  |  |  | DL |
| 1548 | Bacteroidota | Bacteroidia | Sphingobacteriales | Lentimicrobiaceae | Lentimicrobiaceae | DL |
| 1648 | Firmicutes | Clostridia | Peptostreptococcales-Tissierellales | Peptostreptococcales-Tissierellales | Anaerococcus | BG |
| 1656 | Verrucomicrobiota | Verrucomicrobiae | Verrucomicrobiales | Verrucomicrobiaceae | Verrucomicrobium | BG |
| 1663 | Firmicutes | Bacilli | Staphylococcales | Gemellaceae | Gemella | OE |
| 1673 | Proteobacteria | Gammaproteobacteria | Pasteurellales | Pasteurellaceae | Haemophilus | BG |
| 1674 | Bacteroidota | Bacteroidia | Flavobacteriales | Flavobacteriaceae | Flavobacterium | BG |
| 1681 | Proteobacteria | Alphaproteobacteria | Caulobacterales | Caulobacteraceae |  | BG |
| 1691 | Bdellovibrionota | Oligoflexia | Oligoflexales | Oligoflexales | Oligoflexus | BG |
| 1698 | Actinobacteriota | Acidimicrobiia | Microtrichales | Ilumatobacteraceae | CL500-29-marine-group | DL |
| 1705 | Campilobacterota | Campylobacteria | Campylobacterales | Sulfurospirillaceae | Sulfurospirillum | BG |
| 1718 | Proteobacteria | Alphaproteobacteria |  |  |  | DL |
| 1720 | Fusobacteriota | Fusobacteriia | Fusobacteriales | Fusobacteriaceae | Cetobacterium | DL |
| 1753 | Bacteroidota | Bacteroidia | Chitinophagales | Chitinophagaceae |  | ES |
| 1764 | Patescibacteria | Berkelbacteria | Berkelbacteria | Berkelbacteria | Berkelbacteria | DL |
| 1767 | Proteobacteria | Alphaproteobacteria | Rhizobiales | Rhizobiaceae | Allorhizobium-Neorhizobium-Pararhizobium-Rhizobium | BG |
| 1775 | Bacteroidota | Bacteroidia |  |  |  | BG |
| 1789 | Proteobacteria | Alphaproteobacteria |  |  |  | BG |
| 1799 | Proteobacteria | Gammaproteobacteria | Enterobacterales | Enterobacteriaceae |  | BG |
| 1888 | Proteobacteria | Gammaproteobacteria | Burkholderiales | Oxalobacteraceae |  | BG |
| 1915 | Verrucomicrobiota | Verrucomicrobiae | Chthoniobacterales | Chthoniobacteraceae | Chthoniobacter | BG |
| 1920 | Bacteroidota | Bacteroidia | Bacteroidales | Prevotellaceae | Prevotella | BG |
| 1987 | Verrucomicrobiota | Verrucomicrobiae | Chthoniobacterales | Chthoniobacteraceae | Chthoniobacter | OE |
| 2066 | Proteobacteria | Alphaproteobacteria | Sphingomonadales | Sphingomonadaceae |  | BG |
| 2076 | Firmicutes | Bacilli | Bacillales | Bacillaceae | Anoxybacillus | BG |
| 2085 | Proteobacteria | Alphaproteobacteria | Rhizobiales | Beijerinckiaceae | 1174-901-12 | BG |
| 2086 | Proteobacteria | Gammaproteobacteria | Burkholderiales | Comamonadaceae | Variovorax | BG |
| 2093 | Actinobacteriota | Actinobacteria | Micrococcales | Micrococcaceae | Rothia | BG |
| 2110 | Proteobacteria | Gammaproteobacteria | Pseudomonadales | Pseudomonadaceae | Pseudomonas | BG |
| 2125 | Proteobacteria | Gammaproteobacteria | Pseudomonadales | Moraxellaceae | Acinetobacter | BG |
| 2178 | Proteobacteria | Gammaproteobacteria | Burkholderiales | Oxalobacteraceae |  | BG |
